# Core N-DRC components play a crucial role in embryonic development and postnatal organ development

**DOI:** 10.1038/s41419-025-07506-2

**Published:** 2025-03-15

**Authors:** Chuan Ren, Shuya Sun, Jiajie Zhu, Shushu Zhou, Xin Zhang, Shuhui Bian, Ying Wang, Jintao Zhang, Mingxi Liu

**Affiliations:** 1 State Key Laboratory of Reproductive Medicine and Offspring Health, Department of Histology and Embryology, School of Basic Medical Sciences, Nanjing Medical University, Nanjing, China; 2 Department of Reproduction, Nanjing Women and Child’s Healthcare Hospital, Women’s Hospital of Nanjing Medical University, Nanjing, China; 3 State Key Laboratory of Reproductive Medicine and Offspring Health, Clinical Center of Reproductive Medicine, The First Affiliated Hospital of Nanjing Medical University, Nanjing, China

**Keywords:** Ciliogenesis, Infertility

## Abstract

Motile cilia and flagella are evolutionarily conserved organelles, and their defects cause primary ciliary dyskinesia (PCD), a disorder characterized by systemic organ dysfunction. The nexin-dynein regulatory complex (N-DRC) is a crucial structural component of motile cilia and flagella, present across various species from *Chlamydomonas* to humans. Defects in N-DRC components lead to multiple PCD symptoms, including sinusitis and male infertility. However, the phenotypic expression of N-DRC defects varies significantly among individuals, and there has been a lack of systematic study of core N-DRC components in mammals. Utilizing *Drc1-4* and *Drc7* knockout mice, this study systematically reveals the roles and assembly process of core N-DRC components in ependymal cilia, respiratory cilia, and sperm flagella. The findings show that core N-DRC components are crucial for the survival of mice on a purebred genetic background. In mixed genetic background mice, N-DRC defects impair the motility of motile cilia and the stability of flagellar axonemes. Additionally, a novel role of the N-DRC specific component (A-kinase anchoring protein 3) AKAP3 in regulating sperm phosphorylation was discovered. Collectively, our results provide a comprehensive understanding of the core N-DRC components in mammalian cilia and flagella.

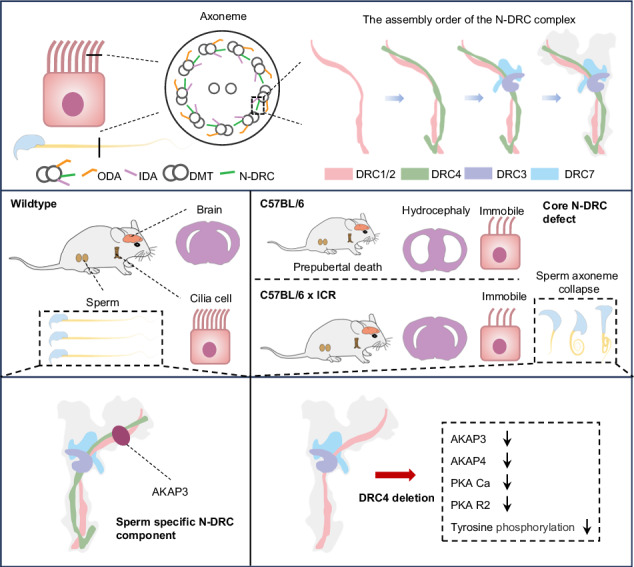

## Introduction

Motile cilia and flagella are evolutionarily conserved organelles involved in sensation and motility, protruding from the surface of many cells, including both prokaryotic and eukaryotic cells [1–3]. In mammals, genetic mutations causing motile cilia dysfunction result in primary ciliary dyskinesia (PCD), a rare inherited disorder primarily characterized by recurrent airway infections and affecting ~1 in 10,000 individuals globally [4–6]. PCD can also cause non-respiratory complications such as left-right laterality, hydrocephalus, and male and female infertility or subfertility [7, 8]. The central structural component of motile cilia and flagella is the axoneme, consisting of nine outer doublet microtubules and a central pair of singlet microtubules, known as the “9+2” arrangement [9–12]. The axoneme also includes accessory structures such as outer dynein arms (ODA), inner dynein arms (IDA), radial spokes (RS), and the N-DRC. Recent advances in cryo-electron tomography and cryo-electron microscopy have provided detailed insights into the axoneme and its accessory structures [13–17].

The N-DRC is a large structural complex composed of two main domains: the base plate and the linker, which connect the A-tubule of one doublet to the B-tubule of the adjacent doublet [18–21]. Previous studies have shown that the N-DRC plays a critical role in regulating flagellar motility, maintaining outer doublet microtubule arrangement, and limiting microtubule sliding in motile axonemes [22, 23]. In *Chlamydomonas*, at least 11 subunits have been identified in the N-DRC, with DRC1, DRC2, and DRC4 serving as core subunits that form a scaffold for other “functional subunits” such as DRC3, DRC5, DRC6, DRC7, DRC8, and DRC11 [13, 16, 20, 24, 25].

Expressional impairments of DRC1, DRC2, and DRC4, core N-DRC structural components, are closely associated with PCD. For instance, a novel DRC1 nonsense variant (Trp432*) was identified in a patient exhibiting bronchiectasis, chronic sinusitis, and significantly reduced nasal nitric oxide production, consistent with a diagnosis of PCD [26]. Additionally, DRC1 defects also result in male infertility, in line with previous research showing that DRC1 mutations cause multiple morphological abnormalities of sperm flagella (MMAF) and male infertility due to failed N-DRC assembly in humans and mice [27]. Mutations in DRC2 and DRC4 have also been found in PCD patients with respiratory and reproductive system symptoms [28–33]. In these studies, patients with DRC deficiencies typically exhibit infertility but may not present with other PCD symptoms. There has been a lack of systematic research on the genetic impact of N-DRC deficiencies in mammals. Although CCDC65 variant lambs and Gas8 mutant mice have been generated, they often suffer from prepubertal death [34, 35]. To date, no DRC2 and DRC4 knockout (KO) animal models have been developed to explore their functional roles in adult mammalian cilia and flagella. Given the potential importance of core N-DRC components, it is imperative to create mammalian models to systematically understand their functions in cilia and flagella.

In this study, we used *Drc1-4* and *Drc7* KO models to systematically reveal the functions and assembly process of core N-DRC components in ependymal cilia, respiratory cilia, and sperm flagella. Unexpectedly, we discovered a novel role of the N-DRC complex in regulating sperm phosphorylation during spermatogenesis.

## Materials/subjects and methods

### Animals

All mice used in this study were housed in a standard animal facility (20–22 °C; 50–70% humidity; 12 h light/dark cycle) with free food and water access. The Institutional Animal Care and Use Committees of Nanjing Medical University approved all studies (Approval No. IACUC-1810020), and all experiments were performed under the guidance of the Animal Ethical and Welfare Committee of Nanjing Medical University. Mouse testes, epididymis and sperms were dissected from 2 months B6(ICR) mice. Mouse brains were dissected from 2-weeks-old C57BL/6 J or 8-weeks-old B6(ICR) mice. Tracheas were dissected from 2 or 8-week-old B6(ICR) mice.

### Cell lines and transfection

HEK293T cells (Enogene) were cultured in DMEM (Gibco) supplemented with 10% Fetal Bovine Serum (Invitrogen) and 1**×** penicillin-streptomycin (Invitrogen) at 37 °C in a humid atmosphere with 5% CO_2_ condition. Regular authentication of cell lines was conducted through short tandem repeat profiling and mycoplasma testing. Transfections of HEK293T cells were performed using Lipofectamine 2000 (Thermo Fisher, MA, USA) according to the manufacturer’s instructions.

*E. coli* DH5a competent cells (Vazyme) were used for molecular cloning, and E. coli strain BL21 (DE3) bacterial strain (Vazyme) was used for protein expressing.

### Generation of *Drc2* and *Drc4* knockout mice by CRISPR/Cas9

KO mice were established as described below. The *Drc2* and *Drc4* KO mice were generated by using two sgRNAs targeted to KO the exon 4 of *Drc2* (5’-GAAGGAGATGCACTACCTACAGG-3’ and 5’-CCCACATGCTCTGGAACT-CCAGC-3’) and the exon 4 of *Drc4* (5’-GTACGAGCACCAGAACAACTTGG-3’ and 5’-CCGCATGTCCTTGCGCAGAGCGC-3’). Following linearization with AgeI, a Cas9 plasmid (Addgene, Watertown, MA, USA) was purified using the MinElute PCR Purification Kit (QIAGEN, Duesseldorf, Germany). And then, the mMESSAGE mMACHINE T7 Ultra Kit (Ambion, Austin, TX, USA) was used to transcribe Cas9 mRNA that was subsequently purified with the RNeasy Mini Kit (QIAGEN, Duesseldorf, Germany) based on the instructions. The Cas9 mRNA (50 ng/μL) and sgRNA (20 ng/μL) were then co-injected into murine zygotes which were transferred into pseudopregnant females. ldentification of new-born mice (7-day-old) was accom-plished through toe clipping, and tissue extraction was carried out using the Mouse Direct PCR kit (Biotool, Shanghai, China). The obtained samples were subjected to amplification utilizing 2xTaq Master Mix (Vazyme) with specified primers (*Drc2*-Forward: 5’-ATGTCATAGTCCTGGTTGTC-3’, *Drc2*-Reverse: 5’-CAGTTCT-GGTTGCCTTGA-3’, *Drc4*-Forward: 5’-TGAGCTGGGCACACACTGGT-3’, *Drc4*-Reverse: 5’-CGCCCCCCATCTTAAGTTCC-3’). The analysis of resulting products was subsequently undertaken through Sanger sequencing.

### Male fertility testing

Three adult *Drc2* or *Drc4* KO male mice (8–12 weeks old) housed with three adult (8–12 weeks old) WT female mice at least 4 months, with WT male mice undergoing the same housing conditions as a control. The next morning, the female’s vaginal orifice was checked to confirm whether they mated. After 19–21 days, we observed whether the female mice have offspring and counted the number of cubs in WT and KO groups.

### Histological analysis

Mouse testes, epididymis and brains were collected from at least three mice for each genotype, then fixed in modified Davidson’s fluid (MDF) for up to 24 h and performed paraffin embedding. Next, fixed tissue were cut as 5 µm thickness sections and mounted on slides, and then performed with H&E staining or periodic acid-schiff (PAS) staining (G1281, Solarbio). For spermatozoa, samples from cauda epididymis were washed in PBS, then fixed in 4% PFA for 30 min and stained in the same way as the mice tissue.

### Western blotting

Proteins were extracted from mice sperm, testes and cultured cells using the lysis buffer (50 mM Tris-HCl pH 8.2, 75 mM NaCl, 8 M urea) containing Protease Inhibitor Cocktail (Roche, Basel, Switzerland) and then mixed with loading buffer, heated at 100 °C for 10 min. These samples were then separated on SDS-PAGE, transferred onto PVDF membranes, blocked with 5% non-fat milk in TBS at room temperature for 1 h, and incubated overnight at 4 °C with relevant primary antibodies. The membranes were rinsed with TBST (TBS+0.1% Tween-20), followed by incubating with corresponding secondary antibodies for 1 h at room temperature. The visualized of detected protein was performed with Enhanced chemiluminescence (ECL) (4600SF, Tanon, Shanghai, China). Primary and secondary antibodies are listed in key resources table.

### Sperm motility analyses

Spermatozoa were extracted from the cauda epididymis, and dispersed in human tubal fluid (HTF, FUJIFILM Irvine Scientific, Japan) containing 10% FBS at 37 °C for 5 min, and then were analyzed by CASA. Hamilton Thorne’s Ceros II system (Hamilton-Thorne Research, Inc., Beverly, MA, USA) was used to dilute and analyze these samples.

### Antibody preparation of DRC1, 2, 3, 4 and DRC7

Production of anti-DRC1, 2, 3, 4 and anti-DRC7 polyclonal antibodies was described previously [36]. The sequence encoding mouse *Drc1* cDNA (aa 1-146), *Drc2* cDNA (aa 1–126), *Drc3* cDNA (aa 182–300), *Drc4* cDNA (aa 1–478) and *Drc7* cDNA (aa 1-292) were cloned and inserted into pET-28a (+) expressing vector, followed by transformation into *E. coli* strain BL21 (DE3), then the fusion proteins were affinity purified with Ni-NTA His Bind Resin (TransGen Biotech, Beijing, China). Two rabbits and four mice were immunized with the fusion protein, yielding antibodies antisera.

### Plasmids

Based on the original CAG plasmid (a gift from the Ikawa laboratory, Osaka University) [37], the plasmids with FLAG and HA tag were constructed, respectively. Full length or truncated *Drc1*, *Drc2*, *Akap3* and *Drc4* CDS sequence were cloned from mouse testis cDNA by PCR and were inserted into pCAG-HA/FLAG plasmid. Based on the original psiCHECK-2 plasmid, the plasmids with luciferase tag were constructed. The initial SV40 promoter and hRluc sequence were deleted and instead SV40-DRC1-FLAG and CMV-DRC2-HA sequence were inserted into plasmids individually or in combination. Finally, these plasmids were confirmed by sequencing.

### Immunofluorescent staining

Mouse testes and brains fixed by MDF performed paraffin embedding and were cut as 5 µm thickness sections and following below procedures. For testicular suspension, fresh testes tissue was gently ground in phosphate-buffered saline (PBS), then stewing for one minute to collect the supernatant. Trachea ciliated cells are dissociated from the inner surface of the trachea with the help of 8 mm medical tracheal brushes. Testicular suspension, mouse spermatozoa and trachea ciliated cells were fixed in 4% PFA for 30 min. After three 10-min washes with PBS, heat-induced antigen retrieval was carried out by boiling the slides in 10 mM citrate buffer (pH 6.0) in a microwave oven for 10 min. Subsequently, the cell smears were washed with PBS three times (10 min/time), blocked with 5% BSA for 1 h, and incubated overnight at 4 °C with relevant primary antibody. After three additional PBST (PBS+0.1% Tween-20) washes, slides were incubated for 2 h with secondary antibodies, counterstained for 5 min with Hoechst 33342 (Invitrogen, Carlsbad, CA, USA), rinsed with PBST, mounted, and imaged with a LSM800 confocal microscope (Carl Zeiss AG, Jena, Germany) and TCS SP8X confocal microscope (Leica Microsystems, Wetzlar, Germany).

### Scanning electron microscopy (SEM)

Mouse tracheas and brains were fixed for 2 h with 2.5% phosphate-buffered glutaraldehyde at 4 °C. Both sample types were then washed with PBS, dehydrated with a chilled ethanol gradient (30%, 50%, 70%, 80%, 90%, and 100%), and subjected to critical point drying with a Lecia EM CPD300 Critical Point Dryer (Wetzlar, Germany). Samples were then attached to appropriate specimen holders and coated with gold particles via the use of an ion sputter coater (EM ACE200, Leica). The images were performed with the Helios G4 CX scanning electron microscope (Thermo Scientific).

### Transmission electron microscopy (TEM)

Fresh brains, trachea and spermatozoa were fixed overnight in 2.5% glutaraldehyde, with subsequent exposure to 2% OsO_4_, and then Araldite for embedding purposes. Ultrathin 80 nm sections staining were done with uranyl acetate and lead citrate, and analysis with an electron microscope (JEM.1010, JEOL).

### Analysis of tracheal ciliary length

Trachea cilia cells were isolated and stained using anti-acetylated α-tubulin antibody and Hoechst as described above. The fluorescence images were photographed with the confocal microscope (Leica TCS SP8X) and the cilia length was determined using LAS X (Leica) software by measuring the length of the ciliary tuft of each cell. At least twenty cells were analyzed in each experimental group.

### Analysis of cilia-driving flow in brain and trachea

The fresh trachea was dissected from 2-weeks-old mice and placed in DMEM supplemented with 10% FBS (Gibco), opened on the dorsal side and fragmented into ~5 mm tissue, followed by transferred to a confocal dish (BDD012035, BIOFIL) to image under a 40× objective (CFI S Plan Flour ELWD NAMC) with an inverted microscope (Eclipse Ti2-U, Nikon). Movies were recorded for 10 s at frame rates of 25 fps. The fresh brain was dissected from 8-weeks-old mice and separated the lateral ventricle and cut it into thin slices in DMEM supplemented with 10% FBS, then transferred to a confocal dish to image under the same condition.

To analyze cilia-generated flow, Beads (1:200, Thermo, FluoSpheres Fluorescent Microspheres 1 µm) were added to a confocal dish and the bead flow was recorded at 23 fps for 6 s using fluorescent microscopy at 24–27°C. Trajectories of beads near the ciliary tuft were analyzed with ImageJ (ImageJ 1.53c, USA).

### Co-immunoprecipitation

HEK293T cells transfected with overexpression plasmids were extracted in RIPA lysis buffer (Beyotime, Shanghai, China) supplemented with proteinase inhibitor. Cell protein lysates were incubated with 50 µl protein A/G magnetic beads (MBL, Chiba, Japan) conjugated HA antibody overnight at 4 °C before taking 50 µl samples as an input. Then, magnetic beads were washed five times with RIPA lysis buffer and boiled for 5 min in SDS loading buffer before SDS/PAGE.

### IP-mass spectrometry

DRC4 was immunoprecipitated from mouse testis using the Pierce crosslink IP kit (26147, Thermo Scientific) with anti-DRC4 antibody described above; IP was performed according to the manufacturer’s instructions. Eluates were precipitated with five volumes of −20 °C pre-chilled acetone followed by trypsin digestion. Trypsin peptide mixture was loaded into the analytical column (Acclaim PepMap C18, 75 μm x 25 cm; Thermo Scientific). The eluted peptides were separated by linear gradient before subsequent analysis. LC-MS/MS analysis was performed on EASY-nanoLC 1000 system (Thermo Scientific) coupled to an Orbitrap Fusion Tribrid mass spectrometer (Thermo Scientific) by a nano spray ion source.

### Quantification and statistical analysis

Data are given as mean ± SD and were compared via two-tailed unpaired Student’s *t*-tests with *α* = 0.05. Where the variance was not similar between the groups that were being statistically compared, a unpaired *t* test with Welch’s correction was used. *p < 0.05, **p < 0.01, ***p < 0.001. Microsoft Excel and GraphPad Prism were utilized for all statistical testing. More than three replicates were conducted to ensure reproducibility. The sample sizes are displayed in the figure legends. Experimental sample size was estimated based on our previous experience performing similar studies in mice. There was no data exclusion.

### Other key resources

This section includes antibodies, reagents and other resources and has been described in detail in Supplementary Table 1.

## Results

### The core N-DRC components are evolutionarily conserved protein and required for murine survival

**Fig. 1:**
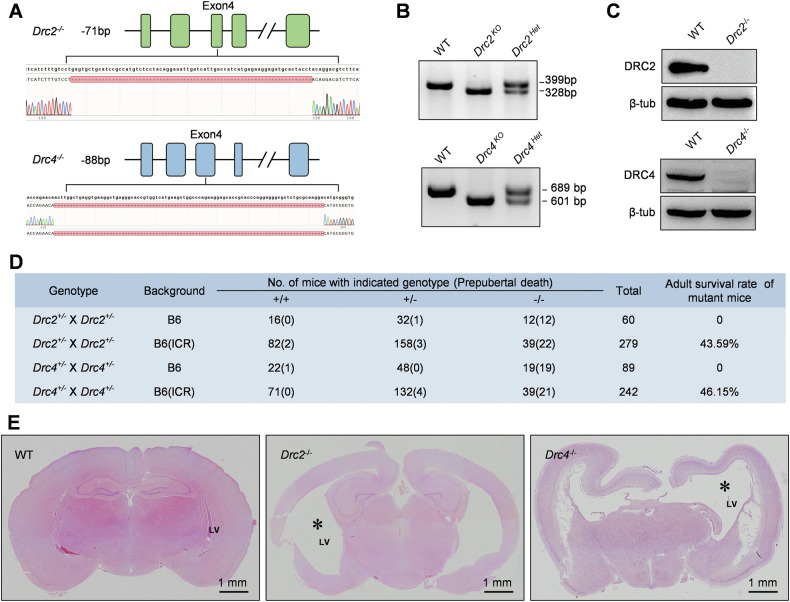
DRC2 and DRC4 are evolutionarily conserved protein and required for murine survival. **A** Knockout strategy of mouse *Drc2* and *Drc4*. Dual sgRNAs were targeted to exon 4 of *Drc2* and *Drc4*, respectively. Sanger sequencing revealed deletion of base pairs fragment. **B** Genotype validation of *Drc2* and *Drc4* using PCR and DNA agarose gel electrophoresis. **C** The expression of DRC2 and DRC4 proteins in the testes of WT and knockout mice was assessed using specific antibodies. Staining for β-tubulin showed no significant differences between WT and knockout mice. **D** The survival rate of mutant mice post-puberty was assessed across different genetic backgrounds. **E** Hematoxylin and Eosin (HE) staining of the cerebrum frontal sections from adult WT, *Drc2*^*−/−*^ *and Drc4*^*−/−*^ mice. The asterisk represents the lateral ventricle (LV).

Motile cilia and flagella are ancient organelles present in many cells, including prokaryotes and eukaryotes, with conserved structures. Protein sequence alignment across species such as *Chlamydomonas*, zebrafish, clawed frog, mouse, rat, human, and bovine reveals that DRC1 [27], DRC2, and DRC4 are highly conserved (Supplementary Fig. 1A, B). To uncover the functions of core N-DRC components in vivo, we used CRISPR/Cas9 to generate stable *Drc2* and *Drc4* KO mice (Fig. 1A–C). Homozygous mutant mice on the C57BL/6 background exhibited low natality and severe postnatal mortality before sexual maturity, with birth rates of 20% (12/60) and 21.35% (19/89) for DRC2 and DRC4 KO mice, respectively (Fig. 1D). All homozygous mutant mice failed to survive to adulthood due to hydrocephaly (Fig. 1E) and this phenotype is consistent with DRC1 mutant mice [27]. Hybridizing heterozygous mice on the C57BL/6 background with wild-type ICR mice, we obtained heterozygous mice on the C57BL6 × ICR background and then these heterozygotes inbred to obtain homozygous KO mice on the C57BL6 × ICR background. This improved adult survival rates to 43.59% (17/39) and 46.15% (18/39) for DRC2 and DRC4 KO mice, respectively (Fig. 1D). Additionally, a small percentage of *Drc1*^*−/−*^ (Supplementary Fig. 3A), *Drc2*^*−/−*^ and *Drc4*^*−/−*^ mice exhibited situs inversus (Supplementary Fig. 1C). These results indicate that core N-DRC components are crucial for murine natality and post-puberty survival.

### Loss of the core N-DRC components affects ciliary behavior in the trachea and brain

**Fig. 2:**
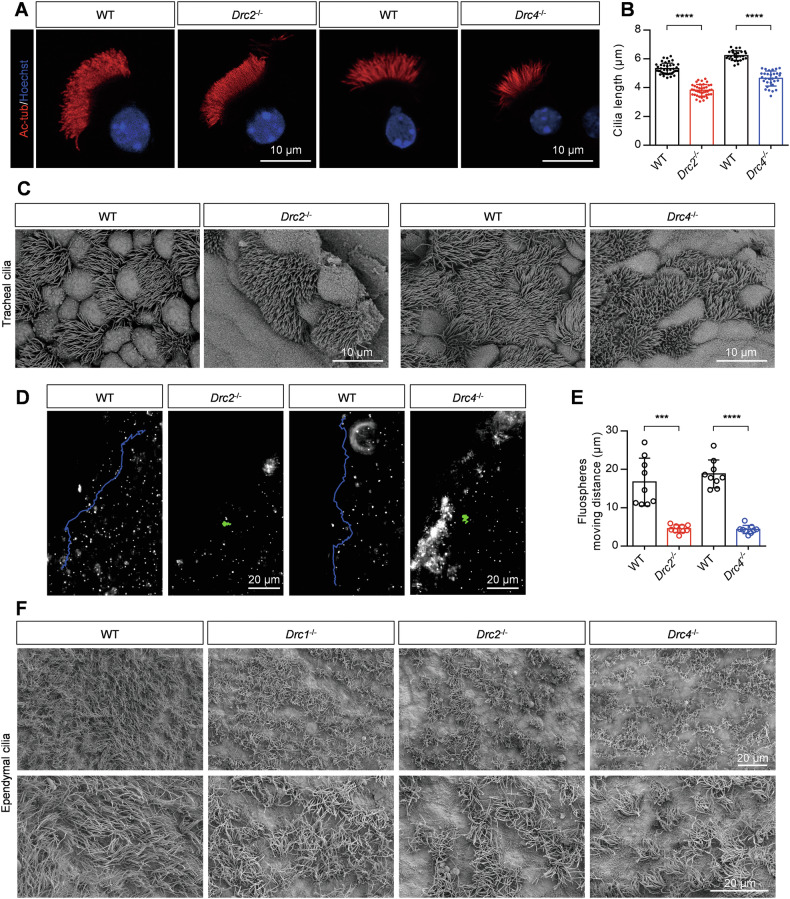
Loss of the core N-DRC components affects ciliary behaviors in brain and trachea. **A** Immunofluorescence analysis of isolated tracheal cilia cells of adult WT, *Drc2*^*−/−*^, and *Drc4*^*−/−*^ mice, using specific acetylated-tubulin antibody against ciliary axoneme (red). Hoechst (blue) stained the nuclei. **B** Evaluation of tracheal cilia length from WT*, Drc2*^*−/−*^, and *Drc4*^*−/−*^ mice (*N* = 3). Error bars denote SD, Student’s *t* test. **C** The tracheal cilia from WT*, Drc2*^*−/−*^, and *Drc4*^*−/−*^ mice, using SEM. **D** Representative fluorescent microspheres motion trajectory driven by liquid flow generated by tracheal cilia cells from WT*, Drc2*^*−/−*^, and *Drc4*^*−/−*^ mice. **E** Evaluation of fluorescent microspheres motion distance (*N* = 3). Error bars denote SD, Student’s *t* test. **F** The ependymal cilia from WT, *Drc1*^*−/−*^, *Drc2*^*−/−*^, and *Drc4*^*−/−*^ mice, using SEM.

Through immunostaining of tracheal ciliary cells and statistical analysis, we found that the cilia length was significantly reduced (Fig. 2A, B). Scanning electron microscopy (SEM) confirmed shorter cilia in the trachea of *Drc2*^*−/−*^ and *Drc4*^*−/−*^ mice compared to controls (Fig. 2C). We next analyzed the ability of cilia to drive fluid flow by adding fluorescent microspheres. In control mice, the fluorescent microspheres moved a longer distance (Supplementary Movies 1 and 2). In contrast, we observed that cilia in *Drc2*^*−/−*^ and *Drc4*^*−/−*^ mice were unable to drive fluid flow, resulting in fluorescent microspheres remaining almost stationary (Fig. 2D, E and Supplementary Movies 3–6). Shorter cilia length and reduced fluid-driving capability were consistent with the results observed in *Drc1*^*−/−*^ mice. Additionally, we investigated ependymal cilia. SEM analysis showed no significant difference in cilia length between *Drc2*^*−/−*^ and *Drc4*^*−/−*^ mice and wild-type mice, but the distribution of ependymal cilia appeared sparser in the mutants (Fig. 2F). Similar results were observed in *Drc1*^*−/−*^ mice (Fig. 2F). We also assessed the ability of ependymal cilia to drive fluid flow. The results showed that, compared to the rapid fluid flow in wild-type mice (Supplementary Movie 7), ependymal cilia in *Drc1*^*−/−*^, *Drc2*^*−/−*^ and *Drc4*^*−/−*^ mice could not drive fluid flow (Supplementary Movies 8–10). These findings suggest that core N-DRC components are essential for proper ciliary function.

### The core N-DRC components are indispensable for sperm flagella assembly and their defections lead to male fertility

**Fig. 3:**
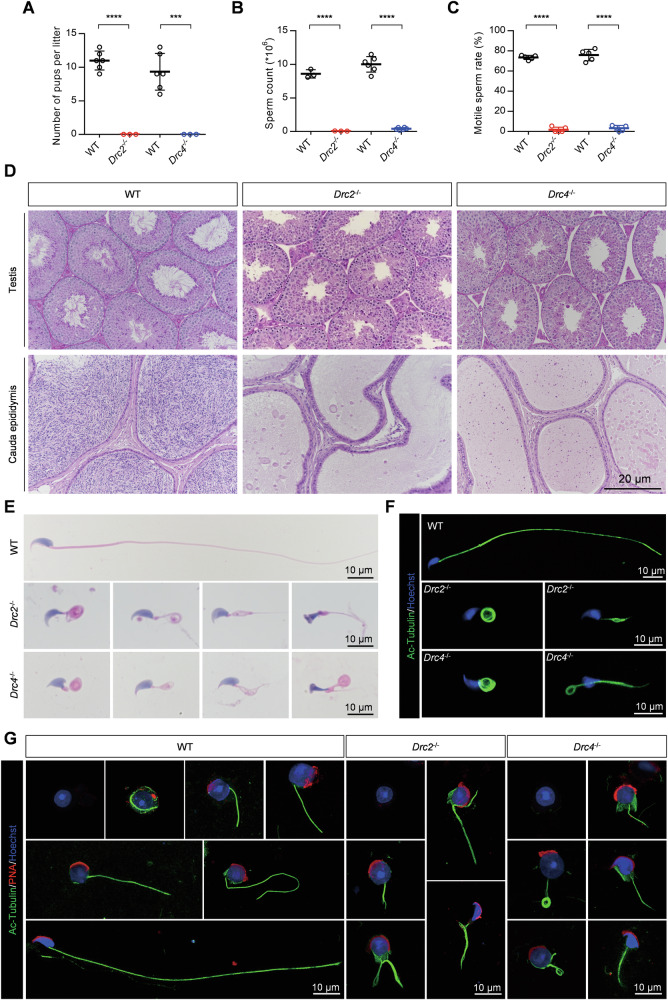
DRC2 and DRC4 are indispensable for sperm flagella assembly and their defections lead to male fertility. **A** Male mice fertility test recorded the number of pups per litter. WT mice, *N* = 6; Knockout mice, *N* = 3. Error bars denote SD, Student’s *t* test. **B** Sperm count in single cauda epididymis (DRC2 group, *N* = 3; DRC4 group, *N* = 6) and **C** sperm motility rate (*N* = 5) from indicated mice. Error bars denote SD, Student’s *t* test. **D** Periodic acid schiff (PAS) staining of testis and HE staining of cauda epididymis from WT, *Drc2*^*−/−*^, and *Drc4*^*−/−*^ mice. **E** HE staining of spermatozoa from WT, *Drc2*^*−/−*^, and *Drc4*^*−/−*^ mice. **F** Immunofluorescence images of spermatozoa from WT, *Drc2*^*−/−*^, and *Drc4*^*−/−*^ mice. Acetylated-tubulin antibody against ciliary axoneme (green). Hoechst (blue) stained the nuclei. **G** Immunofluorescence analysis of testicular suspension samples from WT, *Drc2*^*−/−*^, and *Drc4*^*−/−*^ mice during spermiogenesis. Acetylated-tubulin stained ciliary axoneme (green). PNA stained acrosome (red). Hoechst stained the nuclei (blue).

To analyze the functions of DRC2 and DRC4 in sperm flagella, we assessed male fertility through mating experiments. Individual wild-type or KO male mice were caged with wild-type female mice for 4 months, and the number of offspring per litter was recorded. Despite successful copulations, indicated by the formation of plugs, *Drc2*^*−/−*^ and *Drc4*^*−/−*^ male mice failed to produce any offspring compared to wild-type males (Fig. 3A). No significant differences were found in both the size of the testes and the ratio of testes to body weight (Supplementary Fig. 2A, B). We then evaluated the number of spermatozoa obtained from a single cauda epididymis and found significantly fewer spermatozoa in *Drc2*^*−/−*^ and *Drc4*^*−/−*^ mice (Fig. 3B). Using a computer-assisted sperm analyzer (CASA), we examined the percentage of motile or progressive sperm, revealing almost no motile or forward-moving sperm (Fig. 3C, Supplementary Fig. 2C and Movies 11–13). The dramatically decreased sperm count likely results from defective spermatogenesis. To determine whether a block in spermatogenesis was the cause of the reduced sperm count, we examined spermatogenesis in mutant mice using the PAS staining method. No spermatozoon with normal flagellum in *Drc2*^*−/−*^ and *Drc4*^*−/−*^ mice (Fig. 3D). Furthermore, almost no sperm were observed in the cauda epididymis of *Drc2*^*−/−*^ and *Drc4*^*−/−*^ mice (Fig. 3D).

To investigate the cause of *Drc2*^*−/−*^ and *Drc4*^*−/−*^ mice, we conducted morphological analyses of spermatozoa obtained from the cauda epididymis of wild-type or KO mice using hematoxylin-eosin (H&E) and immunofluorescence staining methods. The sperm from *Drc2*^*−/−*^ and *Drc4*^*−/−*^ mice exhibited multiple morphological abnormalities of the flagella (MMAF) phenotype, including coiled tails, short tails, bent tails, and irregular tails (Fig. 3E, F). This phenotype was similar to KO of non-core N-DRC components, DRC3 [38] and DRC7 [39]. Additionally, we observed aberrant head shapes in the absence of DRC2 or DRC4 compared to controls (Fig. 3E). During mouse spermiogenesis, a transient microtubule structure called the manchette is assembled in step 8 spermatids and disassembled at step 14 [40]. The manchette is associated with sperm head formation, and abnormal head morphology is often related to defective manchette assembly [41–44]. We examined whether the manchette structure was removed normally in *Drc2*^*−/−*^ and *Drc4*^*−/−*^ spermatids using an anti-α-tubulin antibody. The manchette assembly at step 8 was comparable in both wild-type and mutant spermatids (Supplementary Fig. 2D). However, in *Drc4*^*−/−*^ mice, the manchette movement was impaired during steps 9–14, causing delayed caudal movement and unusual manchette location, resulting in a narrow and long-shaped sperm head (Supplementary Fig. 2D).

To further explore the reasons for aberrant sperm morphology in *Drc2*^*−/−*^ and *Drc4*^*−/−*^ mice, we examined the process of flagella formation using immunofluorescence of testicular suspensions (Fig. 3G). Our results revealed that axoneme formation occurred normally throughout spermiogenesis in wild-type spermatids. However, the flagellar axoneme in *Drc2*^*−/−*^ and *Drc4*^*−/−*^ spermatids became disordered early in spermiogenesis and remained so. These findings, along with results from *Drc1*^*−/−*^ mice [27], highlight the importance of core N-DRC components for sperm flagella formation and male fertility.

### Differential ultrastructure between cilium and flagellum in *Drc1*^*−/−*^, *Drc2*^*−/−*^, and *Drc4*^*−/−*^ mice

**Fig. 4:**
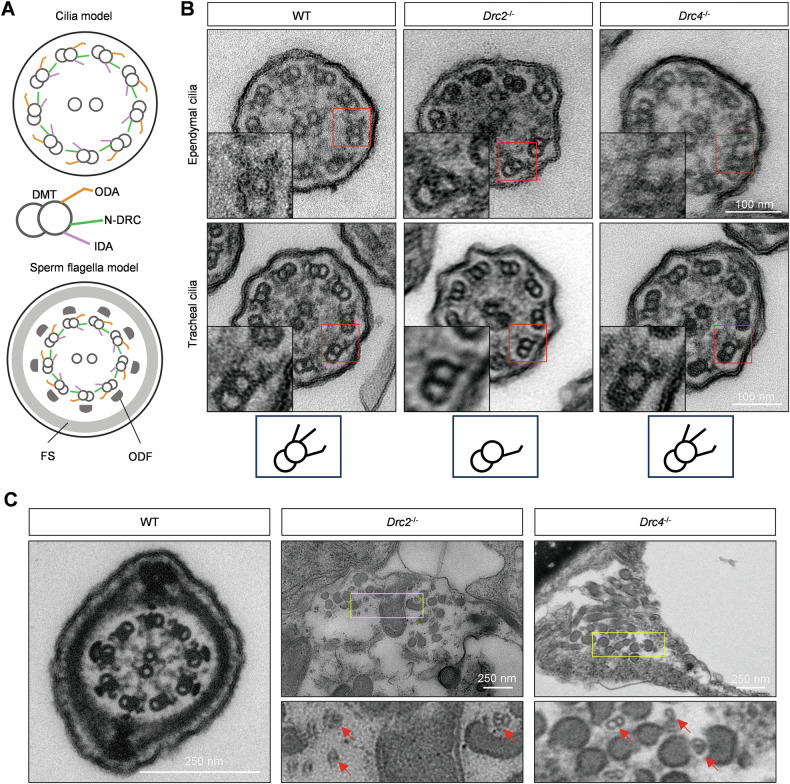
Analysis of the axonemal ultrastructure in cilium and flagellum. **A** Cilium and sperm flagellum axoneme model, comprised of “9+2” microtubule and accessory structures such as RS, ODA, IDA and N-DRC. In sperm, FS and ODF surrounded doublet microtubule. **B** The ultrastructure of ependymal and tracheal cilia cross-section using TEM. The images inside the red rectangle have been enlarged and displayed in the lower left part of each image. The bottom is simple doublet microtubule model corresponding to WT, *Drc2*^*−/−*^, and *Drc4*^*−/−*^ cilia. **C** Sperm structure of WT, *Drc2*^*−/−*^, and *Drc4*^*−/−*^ mice, using TEM. The bottom panel is zoomed image of yellow box. The red arrows indicated scattered doublet microtubule.

The main component of motile cilia and flagella is the axoneme, composed of “9+2” microtubules and accessory structures (Fig. 4A). We explored ultrastructural differences between wild-type and KO mice in cilia and flagella using transmission electron microscopy (TEM). In ependymal and tracheal cilia, a complete axoneme structure, including outer doublet microtubules, ODA, IDA, RS, and N-DRC, was observed in wild-type mice (Fig. 4B). Although cilia from *Drc2*^*−/−*^ maintained normal “9+2” microtubules, they lacked the N-DRC structure and IDA formation (Fig. 4B). Similarly, in *Drc1*^*−/−*^ ependymal and tracheal cilium [27], the N-DRC structure was absent (Supplementary Fig. 3B). In contrast, no obvious differences in axonemal structures were observed in *Drc4*^*−/−*^ mice, and the N-DRC structure was still apparent between adjacent doublet microtubules (Fig.4B).

In sperm flagellar axonemes, an intact “9+2” microtubule structure surrounded by arranged outer dense fibers (ODFs) and the fibrous sheath (FS) was observed in wild-type mice (Fig. 4C). However, in *Drc2*^*−/−*^ and *Drc4*^*−/−*^ mice, the “9+2” sperm axoneme structure was absent, and scattered doublet microtubules and ODFs were detected in the cytoplasm (Fig. 4C). These results indicate that core N-DRC components have different impacts on the ultrastructure of ciliary and flagellar axonemes.

### The N-DRC assembly is impaired in sperm flagella from *Drc2* and *Drc4* knockout mice

**Fig. 5:**
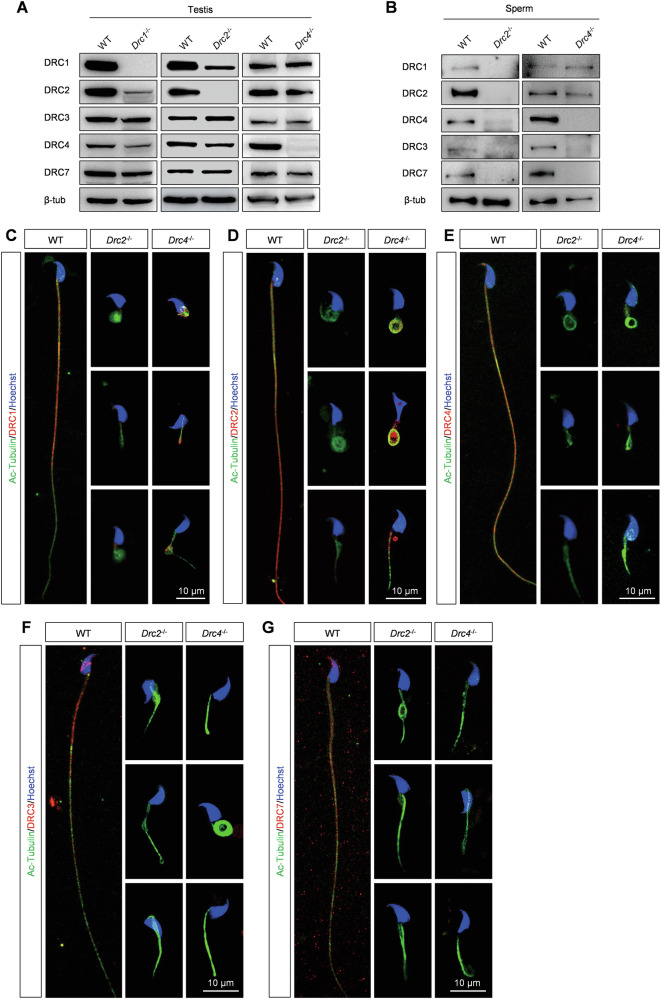
The N-DRC assembly is impaired in *Drc2* and *Drc4* knockout spermatozoa. **A** Different DRCs proteins level in testis from WT, *Drc1*^*−/−*^, *Drc2*^*−/−*^, and *Drc4*^*−/−*^ mice. **B** Different DRCs proteins level in sperm from WT, *Drc2*^*−/−*^, *and Drc4*^*−/−*^ mice. **C**–**G** Immunofluorescence analysis of spermatozoa from WT, *Drc2*^*−/−*^, and *Drc4*^*−/−*^ mice. Specific antibody against DRC1, DRC2, DRC4, DRC3 and DRC7 detected expression level of DRCs (red) in spermatozoa of WT, *Drc2*^*−/−*^, and *Drc4*^*−/−*^ mice. Ac-tubulin (green) stained ciliary axoneme. Hoechst (blue) stained the nuclei.

We explored the effects of DRC2 and DRC4 loss on N-DRC assembly in sperm flagella. Western blotting analysis from testis extracts revealed that all DRC components were expressed in both wild-type and KO mice, except for the deleted one (Fig. 5A). However, in the absence of DRC2, the expression of DRC1, DRC3, DRC4 and DRC7 was also absent in spermatozoa, confirmed by western blotting and immunostaining analysis (Fig. 5B–G). When DRC4 was knocked out, DRC1 and DRC2 were expressed normally in spermatozoa but partially failed to assemble onto flagella (Fig. 5B–E). Similarly, DRC3 and DRC7 were not expressed in *Drc4*^*−/−*^ spermatozoa, consistent with immunostaining analysis (Fig. 5B, F, G).

Moreover, we found that the expression of DRC2 decreased significantly in *Drc1*^*−/−*^ testis, and conversely, the level of DRC1 was lower than in control testes in *Drc2*^*−/−*^ mice (Fig. 5A). Recent structural studies have shown that DRC1 and DRC2 function as a heterodimer [13, 16]. Thus, we hypothesized that DRC1 and DRC2 are more stable in their heterodimeric state than as monomers. By constructing plasmids containing luciferase as a loading control (Supplementary Fig. 4B), we found no significant increase in DRC1 and DRC2 expression in co-transfected HEK293T cells compared to individually transfected cells, whether or not cycloheximide (CHX), a protein synthesis inhibitor, was added (Supplementary Fig. 4A, B). DRC1 or DRC2 in monomeric form may be cleared in a specific way in the testes.

### The N-DRC structure assemble into axoneme in the order of DRC1/2, DRC4, DRC3 and DRC7 in murine cilia

**Fig. 6:**
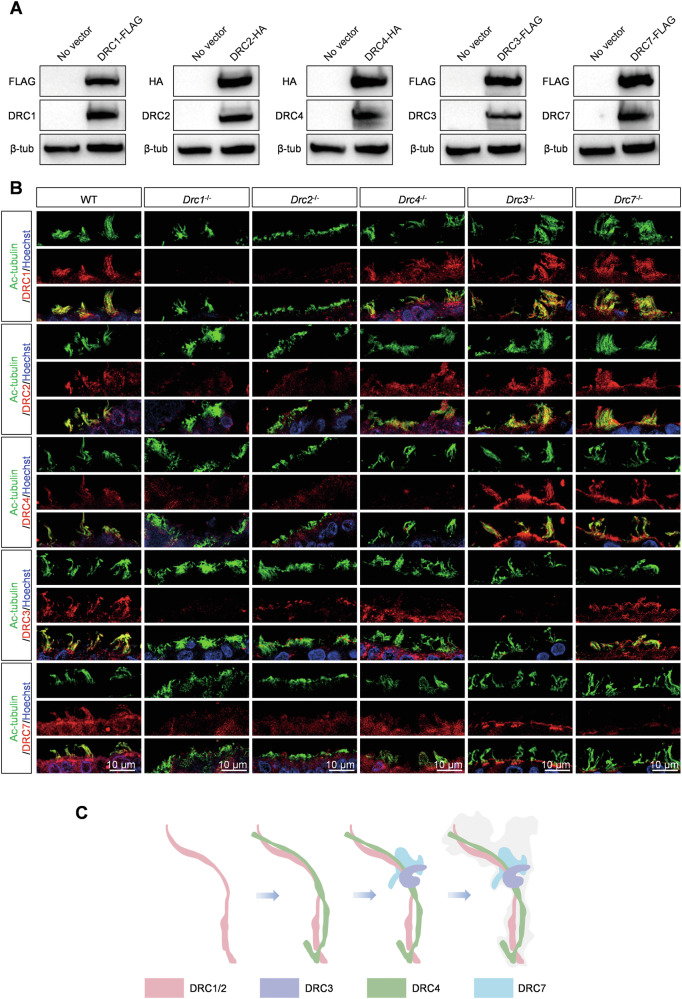
DRC1 and DRC2 are the most core components, followed by DRC4, and then DRC3, and finally DRC7 for the N-DRC structure. **A** Antibody specificity detection of DRC1, DRC2, DRC3, DRC4 and DRC7 through western blots of HEK293T cells transiently expressing DRC1/2/3/4/7-FLAG or HA. FLAG or HA tag antibodies as positive control. **B** Immunofluorescence analysis of ependymal cilia from WT, *Drc1*^*−/−*^, *Drc2*^*−/−*^*, Drc4*^*−/−*^, *Drc3*^*−/−*^, and *Drc7*^*−/−*^ mice. Specific antibody against DRC1, DRC2, DRC4, DRC3 and DRC7 detected expression level of DRCs (red) in ependymal cilia of WT, *Drc1*^*−/−*^, *Drc2*^*−/−*^*, Drc4*^*−/−*^, *Drc3*^*−/−*^, and *Drc7*^*−/−*^ mice. Ac-tubulin (green) stained ciliary axoneme. Hoechst (blue) stained the nuclei. **C** The N-DRC complex assembly pattern.

The N-DRC is a large structural complex comprising at least 11 subunits. We investigated the impact of one DRC component deficiency on the expression of other DRCs in cilia. For the reliability of the results, we first tested the specificity of the antibodies of DRC1/2/3/4/7 through HEK293T cells overexpression experiments (Fig. 6A). When the core components DRC1/DRC2 were deleted, no signals of DRC1-4 and DRC7 were detected in ependymal cilia (Fig. 6B). However, the loss of another core component, DRC4, did not affect the expression of DRC1 and DRC2 in cilia, whereas DRC3 and DRC7 failed to assemble into cilia (Fig. 6B). Hence, we speculated that DRC1 and DRC2 are assembled first, followed by DRC4, and finally DRC3 and DRC7 are assembled into cilia. Introducing *Drc3*^*−/−*^ and *Drc7*^*−/−*^ mice (Supplementary Fig. 3A), our immunostaining analysis demonstrated that DRC3 only affected the assembly of DRC7 into cilia, while DRC1, DRC2, and DRC4 were expressed normally, indicating that DRC3 assembles before DRC7 (Fig. 6B). Immunostaining analysis of tracheal cilia recapitulated the results of ependymal cilia (Supplementary Fig. 5A). These results showed that DRC1 and DRC2 are the most critical components, followed by DRC4, DRC3, and finally DRC7 for the N-DRC structure assembly (Fig. 6C).

### DRC4 interacts with AKAP3 to regulate PKA activity during sperm capacitation

**Fig. 7:**
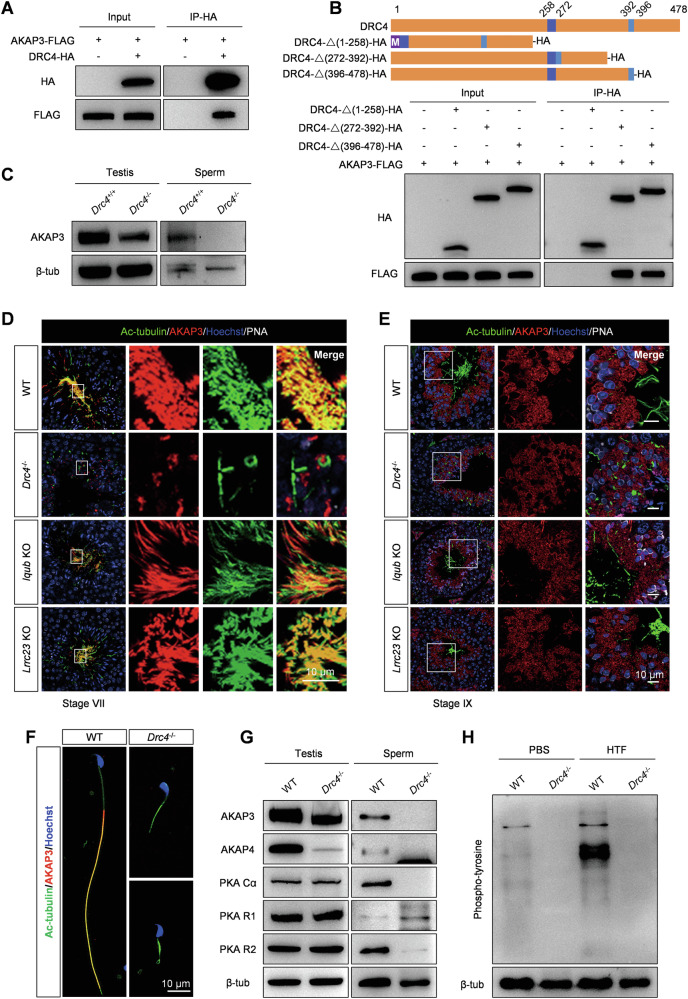
DRC4 interacts with AKAP3 to regulate PKA activity during sperm capacitation. **A** DRC4 interaction with AKAP3 was assessed by co-IP. Input: whole cell lysate from transfected cells. DRC4 interacted with AKAP3 in HEK293T cells. **B** The top part showed the truncated strategy of DRC4 protein. The bottom panel showed the co-IP analysis of truncated DRC4 and AKAP3. M, methionine. **C** Evaluation of AKAP3 protein in testis and sperm from WT and *Drc4*^*−/−*^ mice, using western blotting. **D**, **E** Immunofluorescence analysis of testis cross-section of WT, *Drc4*^*−/−*^, *Iqub*^*−/−*^, and *Lrrc23*^*−/−*^ mice at stage VII and stage IX, using antibodies to evaluate the expression of Ac-tubulin (green), AKAP3 (red) in testis. PNA stained acrosome (white). Hoechst stained the nuclei (blue). **F** Immunofluorescence analysis of spermatozoa from WT and *Drc4*^*−/−*^ mice. Specific antibody against AKAP3 (red) in spermatozoa of WT and *Drc4*^*−/−*^ mice. Ac-tubulin (green) stained flagellar axoneme. Hoechst (blue) stained the nuclei. **G** Measured levels of AKAP3, AKAP4, PKA Ca, PKA R1, and PKA R2 in testis and sperm from WT and *Drc4*^*−/−*^ mice. **H** Phospho-tyrosine level in sperm from WT and *Drc4*^*−/−*^ mice before (in PBS) and after capacitation (in HTF).

By performing immunoprecipitation (IP)-mass spectrometry of DRC4 in mouse testes, we confirmed that murine DRC4 interacts with AKAP3 (Table 1), which regulates PKA functions by forming a binding site for the regulatory subunits of PKA [45, 46]. Additionally, we also conducted IP-MS for DRC2 and DRC1, but did not find any interaction with AKAP3. Our results determined the specific expression of AKAP3 in the testes (Supplementary Fig. 6A). Previous studies have confirmed that a lack of AKAP3 induces asthenozoospermia and male infertility [47, 48]. Here, we first conducted a co-IP experiment in HEK293T cells and demonstrated the interaction between DRC4 and AKAP3 in vitro (Fig. 7A). To determine which sequence of the DRC4 protein is responsible for binding AKAP3, we constructed truncated DRC4 plasmids, deleting amino acids 1-258, 272-392, or 396-478, respectively (Fig. 7B). Our co-IP results showed that when the first 258 amino acids were removed, the truncated DRC4 could not pull down AKAP3, indicating this sequence as the crucial site for interacting with AKAP3 (Fig. 7B). To understand the impact of *Drc4* KO on the expression of AKAP3, we detected protein levels in testes and sperm. Western blotting revealed that the loss of DRC4 slightly decreased AKAP3 expression in the testes, and unexpectedly, no AKAP3 band was detected in sperm extracts (Fig. 7C). Immunofluorescence analysis of testicular cross-sections indicated that during spermiogenesis, AKAP3 failed to assemble onto sperm flagella in *Drc4* KO mice, although its synthesis was not influenced in the spermatid cytoplasm compared to controls (Fig. 7D, E). However, both the location and synthesis of AKAP3 were normal when RS1 (IQUB) [49] or RS3 (LRRC23) [50] structure were deleted (Fig. 7D, E). Immunofluorescence analysis of spermatozoa further validated AKAP3 could not assemble to sperm flagellum (Fig. 7F). Altogether, the new N-DRC component AKAP3, specifically expressed in the testes, was identified as a downstream binding protein of DRC4.

**Table 1:** Testicular interaction components identified by mass spectrometry.

Gene names	MS/MS count Drc4 WT-1	MS/MS count Drc4 WT-2	MS/MS count Drc4 KO-1	MS/MS count Drc4 KO-2	MS/MS count	Protein names
Gas8	9	12	0	0	21	Growth arrest-specific protein 8
Akap3	1	1	0	0	2	A-kinase anchor protein 3

The immunoprecipitation analysis of testicular interaction components from the WT and *Drc4*^−/−^ mice. Components were identified via mass spectrometry.

Structural defects in AKAP3 mutant sperm could result in the displacement of PKA subunits [47]. We examined proteins extracted from testes and sperm in *Drc4* KO mice. Western blot analysis indicated that in *Drc4* null sperm, both PKA catalytic subunit (Cα) and PKA regulatory subunit (R2) subunits were not detected, whereas PKA R1 appeared to be increased. We also found that AKAP4 was absent in mutant sperm and had extremely low expression in testis lysates (Fig. 7G). During capacitation, a global up-regulation in phospho-tyrosine expression of sperm proteins is a hallmark, and PKA activity leads to increased tyrosine phosphorylation by activating tyrosine kinases [51, 52]. We tested the expression level of phospho-tyrosine before and after sperm capacitation with a specific antibody against tyrosine phosphorylation. The results revealed that tyrosine-phosphorylated proteins extracted from capacitated (HTF) sperm increased compared to basal (PBS) sperm in wild-type mice (Fig. 7H). By contrast, no tyrosine-phosphorylated band was detected in *Drc4* KO sperm under both PBS and HTF conditions (Fig. 7H). We observed the localization of AKAP3 in motile cilia/flagella and found that it is present only in sperm flagella (Supplementary Fig. 6A). These results suggest that the N-DRC complex in sperm axonemes has a unique role in sperm tyrosine phosphorylation, which is distinct from its function in motile cilia.

## Discussion

### Core N-DRC components play a crucial role in embryonic development and postnatal organ development

In this study, we systematically explored the functions and assembly order of the evolutionarily conserved core N-DRC components in mammals using *Drc1-4* and *Drc7* KO models. To our knowledge, there is currently no *Drc2* KO animal model for functional research. Previous study by Wesley R. Lewis et al. reported a *Drc4* gene edited mice. However, they inserted a β-geo cassette in intron 7 of the *Drc4* gene, which led to mutant mice translating a large fusion protein including DRC4 and β-geo. In contrast, utilizing CRISPR/Cas9 we generated *Drc4*^*−/−*^ mice by deleting 88-bp in exon 4 and confirmed it by PCR and western blots. Additionally, their mice survived for up to 21 days so that they only reported hydrocephalic and situs inversus phenotypes. In this study, we have successfully obtained adult *Drc2* and *Drc4* KO mice to systematically investigated their structure and function in cilia and flagella.

We found that KO mice for the core N-DRC components exhibited prepubertal lethality on an inbred genetic background. In a mixed genetic background, although more than half of the mice died due to severe hydrocephalus, we observed some healthy surviving mice. This could be understood as a heterosis in tolerance to motile cilia defects. Moreover, we found that the genotypes of the newborn offspring of these gene-edited heterozygous mice did not follow Mendelian inheritance laws, suggesting that the loss of DRC1/2/4 partially caused embryonic lethality.

Further research showed that the core N-DRC components are essential for ciliary and flagellar function. Their deficiencies led to shorter respiratory cilia, sparser ependymal cilia, impaired fluid flow, disrupted sperm flagellar formation, reduced sperm count, and male infertility. Consistent with *Drc1*^−/−^ mice, abnormal head shapes in *Drc2*^*−/−*^ and *Drc4*^*−/−*^ spermatozoa were mainly attributed to abnormal manchette removal. As PCD-related genes, *Drc1*^*−/−*^, *Drc2*^*−/−*^, and *Drc4*^*−/−*^ mice also exhibited situs inversus, which implicates nodal cilia [53]. Further structural evidence of nodal cilia is needed to confirm these conclusions.

Although DRC1, DRC2, and DRC4 are considered core components of the N-DRC, our TEM studies of axoneme structure in *Drc1*^*−/−*^, *Drc2*^*−/−*^, and *Drc4*^*−/−*^ ependymal and tracheal cilia revealed differences among them. Compared to controls, the N-DRC structure was absent in *Drc1*^*−/−*^ and *Drc2*^*−/−*^ cilia but intact in *Drc4*^*−/−*^ cilia. Additionally, IDAs were missing in *Drc2*^*−/−*^ cilia, a phenotype also reported in patients harboring CCDC65/DRC2 mutations [28]. In another study of spermatozoa from a patient with a CCDC65 variant [32], the expression of DNALI1, an IDA component, was significantly decreased. These data imply a relationship worth further exploration between DRC2 and IDA. Unlike ciliary ultrastructure, we observed extremely disorganized axonemal structures in *Drc2*^*−/−*^ and *Drc4*^*−/−*^ sperm flagella, with hardly any doublet microtubules. This difference between cilia and flagella has also been reported in other DRCs such as DRC1, DRC3, DRC5, and DRC7 [27, 38, 39, 54]. Possible explanations include the greater length of sperm flagella compared to cilia and the presence of more accessory structures surrounding the flagellar axoneme, such as ODF, FS, and MS. Another possibility is the differential protein composition between ciliary and flagellar axonemes [55], which may maintain cilia stability.

### N-DRC component assembly and tissue-specific components

Immunostaining analysis of ependymal and tracheal cilia from *Drcs* KO mice revealed that DRC1/2 are required for the complete N-DRC assembly, consistent with TEM results showing the absence of the N-DRC structure in *Drc1*^*−/−*^ and *Drc2*^*−/−*^ cilia. DRC4 is necessary for DRC3/7 assembly, and DRC3 is required for DRC7 assembly onto the axoneme. Thus, among core N-DRC components, DRC1 and DRC2 are more crucial than DRC4 for sustaining the N-DRC structure in cilia. Recent studies suggest that the N-DRC assembles onto the axoneme through the stepwise addition of individual proteins or smaller subcomplexes onto the DMTs [56]. Therefore, we hypothesize that the core components DRC1 and DRC2 first assemble onto the ciliary axoneme as a subcomplex, given their roles as a heterodimer [13, 16], followed by DRC4, DRC3, and finally DRC7 (Fig. 6C). More research is needed to confirm these deductions. Given the prominent decrease in DRC1 expression in DRC2-null testis and vice versa, and the interaction between DRC1 and DRC2, we speculate that DRC1 and DRC2 are more stable as a heterodimer, with redundant DRC1 or DRC2 in germ cells eliminated to maintain a balance. DRC1 or DRC2 in monomeric form may affect sperm flagella formation by binding to microtubule proteins, damaging microtubule organization, or leading to aberrant N-DRC structure or location.

Our IP-MS results in the testis showed that DRC4 interacts with AKAP3, with the first 258 amino acids of DRC4 necessary for this interaction. Loss of DRC4 disrupted PKA structures and functions in testis and sperm, but the deletion of RS components did not affect PKA assembly onto sperm flagella. Based on our knowledge, there has been no research on the relationship between the N-DRC and sperm tyrosine phosphorylation. Our findings indicated that AKAP3 is an N-DRC specific component in sperm flagella. A recent study showed that the N-DRC transmits a mechanical signal between dynein arms or neighboring DMTs through physical links [16]. DRC4 appears to form a physical connection with AKAP3 to modulate PKA activity.

In summary, our results indicate that core N-DRC components are essential for ciliary and flagellar structures and functions. Their defects cause situs inversus, hydrocephalus, and male infertility, emphasizing the importance of DRC1 and DRC2 for N-DRC assembly onto the ciliary axoneme and proposing an N-DRC assembly model. We also propose a novel function of the N-DRC in sperm tyrosine phosphorylation. These findings highlight the vital role of core N-DRC components in mammals.

## Supplementary information

Supplementary information

Supplementary movies

Full and uncropped western blots

## Data Availability

All data are available in the main text or in the supporting information materials. Any material requests and information should be directed to the lead contact. Any additional information required to reanalyze the data reported in this work paper is available from the lead contact upon request.
