# Supplementary material for: Core N-DRC components play a crucial role in embryonic development and postnatal organ development
Source: Cell Death Dis. 2025 Mar 15;16(1):176. doi: 10.1038/s41419-025-07506-2 (PMC11910659; doi:10.1038/s41419-025-07506-2)
Supplement: Supplementary file 1 — Supplementary information [file 41419_2025_7506_MOESM1_ESM.docx]

**SUPPLEMENTARY INFORMATION**

**Core N-DRC Components Play a Crucial Role in Embryonic Development and Postnatal Organ Development**

Chuan Ren, Shuya Sun, Jiajie Zhu, Shushu Zhou, Xin Zhang, Shuhui Bian, Ying Wang, Jintao Zhang, and Mingxi Liu


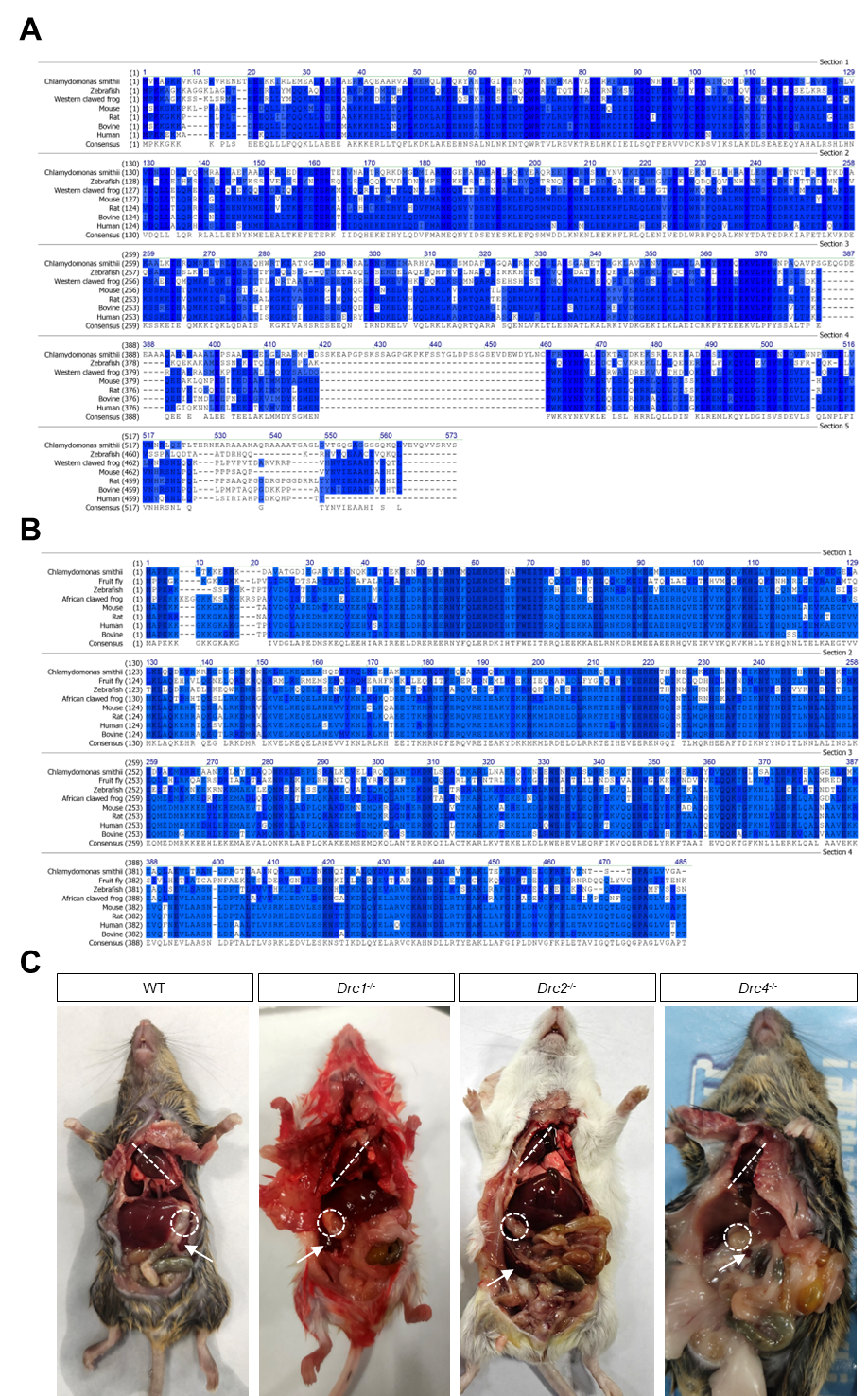


**Supplementary Figure 1. DRC2 and DRC4 are evolutionarily conserved protein****.**

**A-B** The DRC2 and DRC4 protein sequence homology in various organisms. Dark blue background represents identical residues in all species, mid-blue background represents conserved residues and light blue background shows weakly similar residues. **C** Compared to WT, *Drc1^-/-^*, *Drc2^-/-^* *and Drc4^-/-^* mice presented with a laterality defect, including *situs inversus* and heterotaxy. Dotted line showed the direction of cardiac apex. Circular dashed line indicated stomach. Arrow indicated spleen.


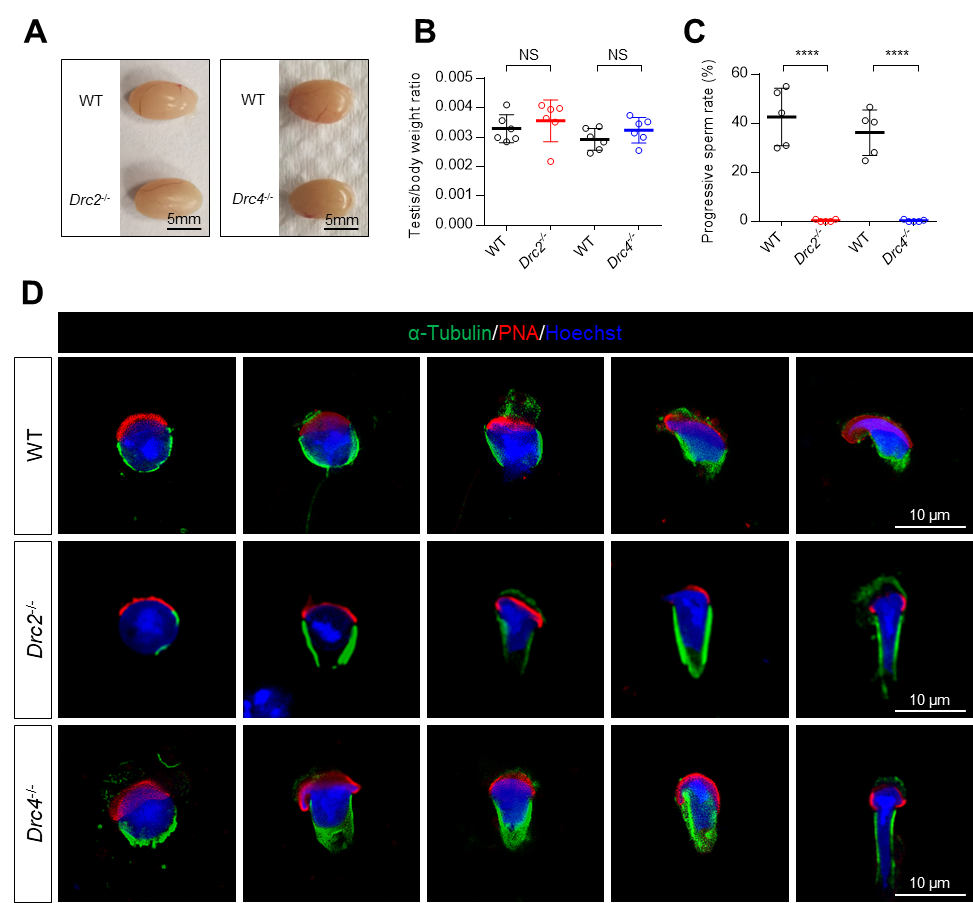


**Supplementary Figure 2. DRC2 and DRC4 are indispensable for sperm flagella assembly and their defections lead to male fertility.**

**A** Testes from WT, *Drc2^-/-^* *and Drc4^-/-^* mice. **B** Evaluation of the weight’s ratio of the testes/body from WT, *Drc2^-/-^* *and Drc4^-/-^* mice (N=6). Error bars denote SD, Student’s t test. **C** The mean percentages of quantification of progressively motile spermatozoa from WT, *Drc2^-/-^* *and Drc4^-/-^* mice (N=5). Error bars denote SD, Student’s t test. **D** Immunofluorescence staining for α-tubulin (green) and PNA (red) in WT, *Drc2^-/-^ and Drc4^-/-^* mice. α-Tubulin stained the manchette microtubules in elongating spermatids. Hoechst (blue) stained the nuclei.

**Supplementary Figure 3. Loss of DRC1 did not impair ‘9+2’ structure.**

**A** Knockout strategy of the mouse *Drc1*, *Drc3* and *Drc7*. DRC1, DRC3 and DRC7 protein expression within WT and knockout mice testis using specific antibodies. β-tubulin staining showing no significant difference between WT and knockout mice. **B** Ependymal cilia cross-section from WT and *Drc1^-/-^* mice, using TEM.


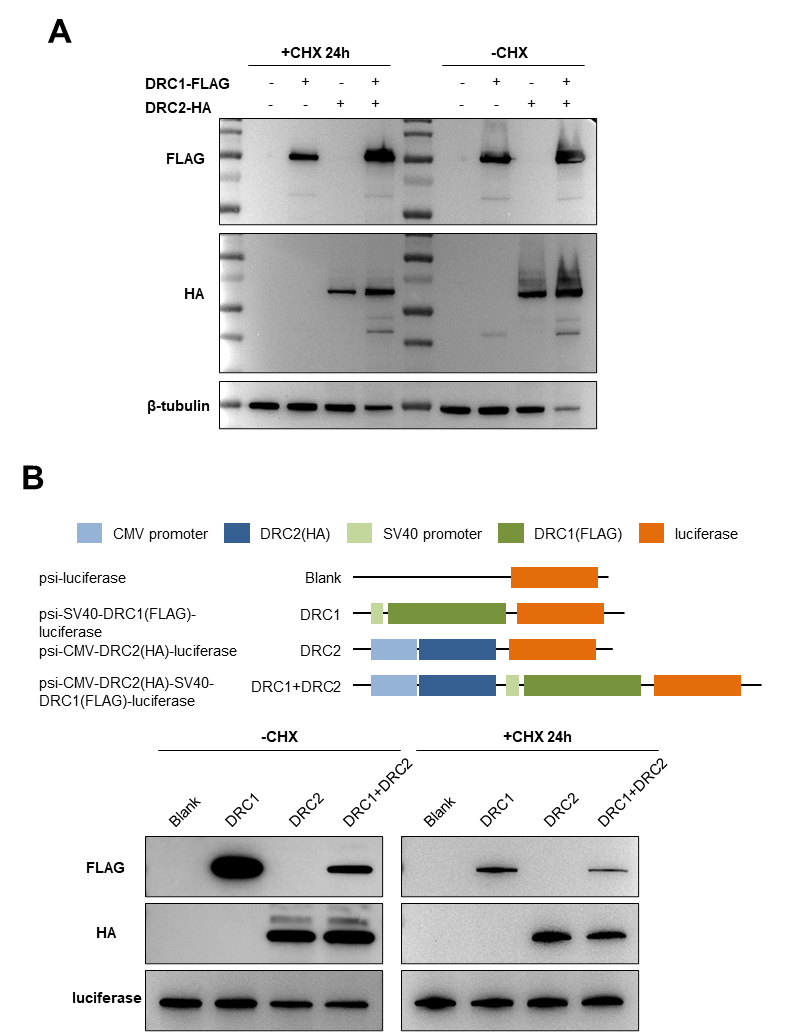


**Supplementary Figure 4. The expression of DRC1 and DRC2 in transfected HEK293T cells.**

**A** The expression levels of DRC1 and DRC2 in transfected HEK293T cells, supplement with CHX or not. β-tubulin as a loading control. **B** The top indicated the construction strategy of plasmids of DRC1, DRC2 and DRC1+DRC2 based on the plasmid “psi-luciferase”. The bottom showed the expression levels of DRC1 and DRC2 in transfected HEK293T cells, supplement with CHX or not. Luciferase as a loading control.


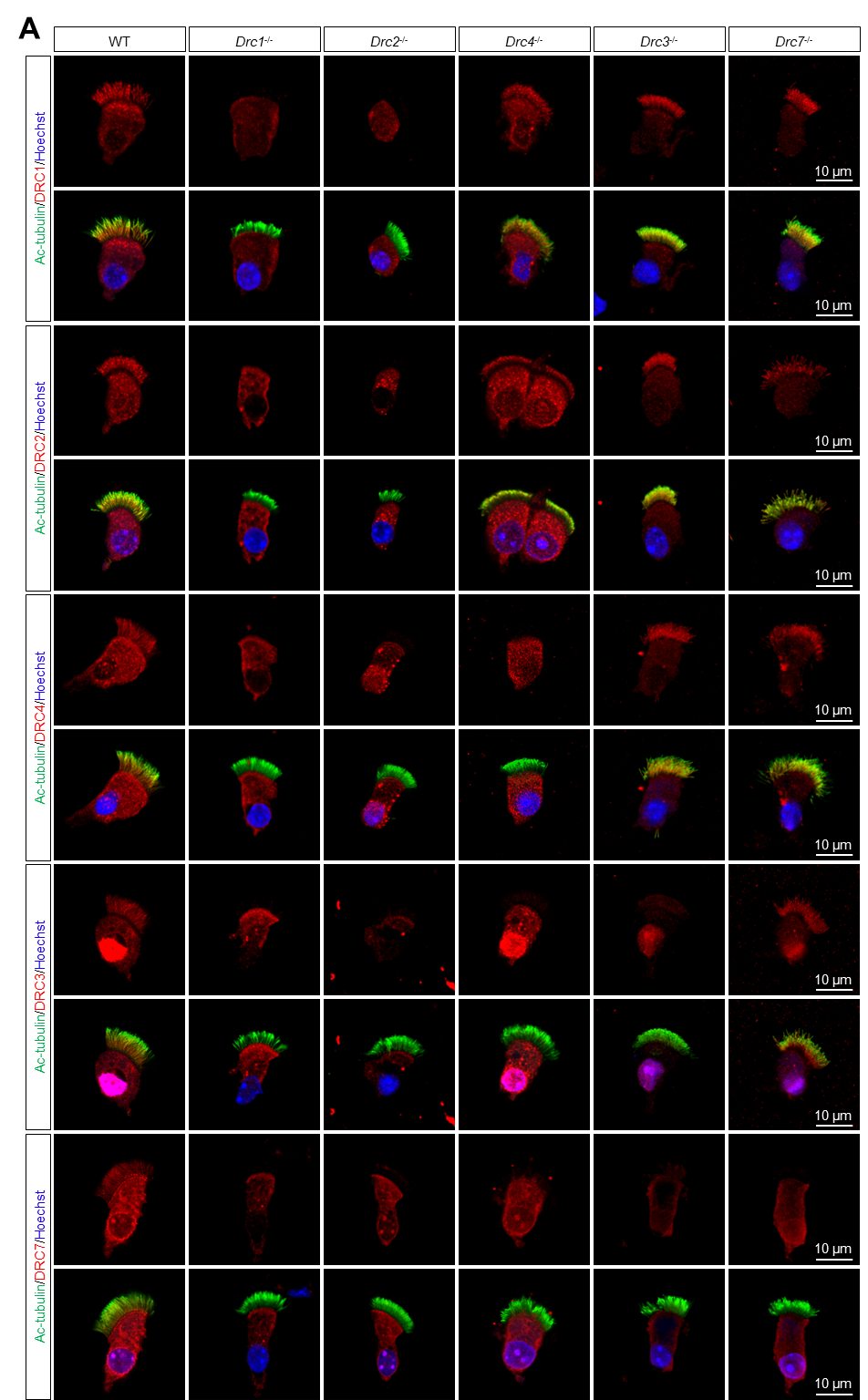


**Supplementary Figure 5. DRC1 and DRC2 are the most core components for the N-DRC assembly.**

**A** Immunofluorescence analysis of tracheal cilia from WT, *Drc1^-/-^*, *Drc2^-/-^, Drc4^-/-^*, *Drc3^-/-^* and *Drc7^-/-^* mice. Specific antibody against DRC1, DRC2, DRC4, DRC3 and DRC7 detected expression level of DRCs (red) in tracheal cilia of WT, *Drc1^-/-^*, *Drc2^-/-^, Drc4^-/-^*, *Drc3^-/-^* and *Drc7^-/-^* mice. Ac-tubulin (green) stained ciliary axoneme. Hoechst (blue) stained the nuclei.


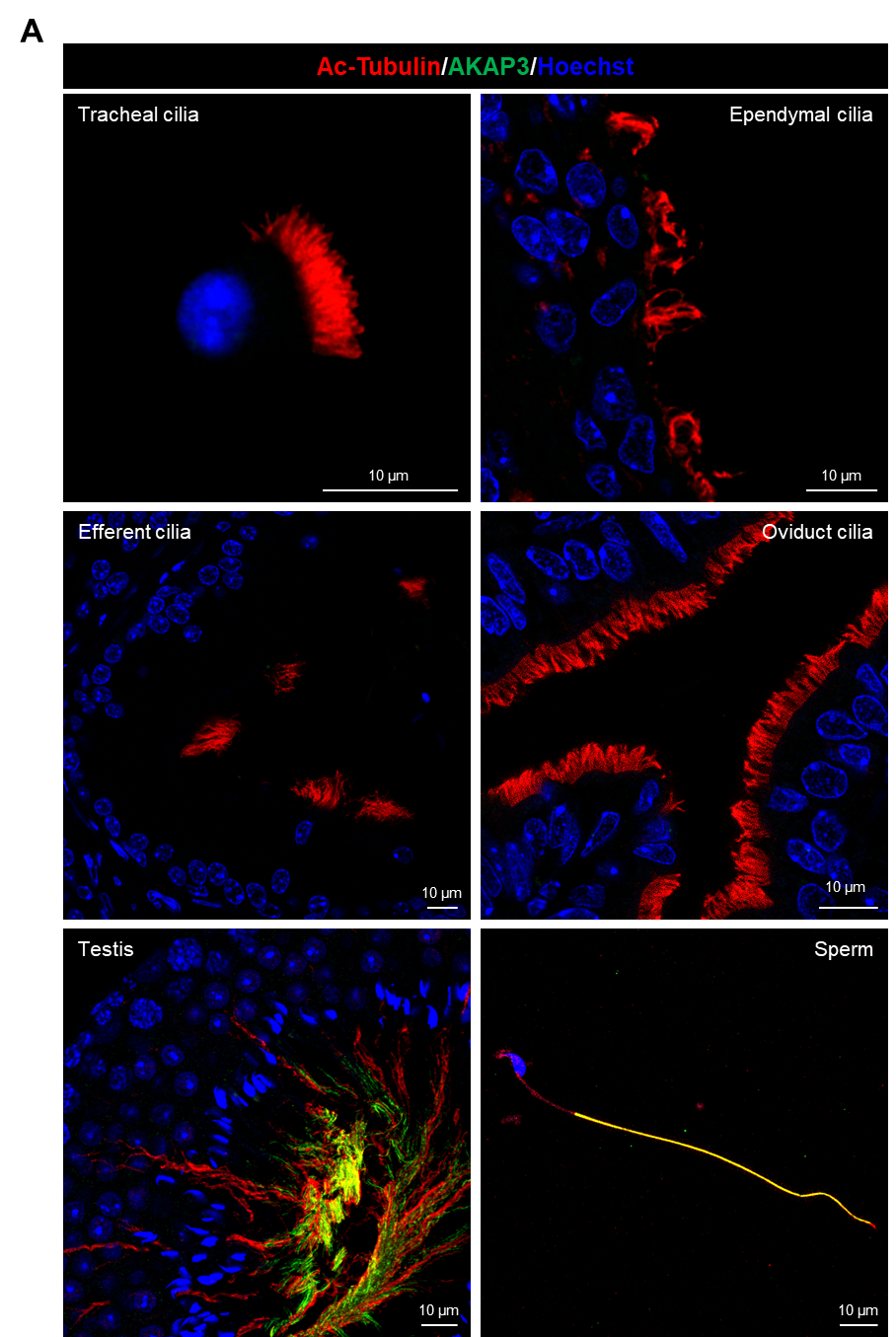


**Supplementary Figure 6. AKAP3 is specifically expressed in sperm.**

**A** Immunofluorescence staining for Ac-tubulin (red) and AKAP3 (green) in WT different tissues as shown in the figures. AKAP3 only expressed in sperm flagella. Ac-tubulin stained ciliary or flagellar axoneme. Hoechst (blue) stained the nuclei.

**Supplementary Movies 1-2.** The beating of tracheal cilia and generating fluid flow in WT mice.

**Supplementary Movies 3-4.** The beating of tracheal cilia and generating fluid flow in *Drc2^-/-^* mice.

**Supplementary Movies 5-6.** The beating of tracheal cilia and generating fluid flow in *Drc4^-/-^* mice.

**Supplementary Movie 7.** The beating of ependymal cilia in WT mice.

**Supplementary Movie 8.** The beating of ependymal cilia in *Drc1^-/-^* mice.

**Supplementary Movie 9.** The beating of ependymal cilia in *Drc2^-/-^* mice.

**Supplementary Movie 10.** The beating of ependymal cilia in *Drc4^-/-^* mice.

**Supplementary Movie 11.** Motility and movement of the sperm from the caput epididymis of WT male mice.

**Supplementary Movie 12.** Motility and movement of the sperm from the caput epididymis of *Drc2^-/-^* male mice.

**Supplementary Movie 13.** Motility and movement of the sperm from the caput epididymis of *Drc4^-/-^* male mice.

**Supplementary Table 1.** Key materials or subjects.

| REAGENT or RESOURCE | SOURCE | IDENTIFIER |
| --- | --- | --- |
| **Antibodies** | | |
| Mouse anti-β-tubulin | Abclonal | AC021 |
| Mouse anti-acetylated tubulin | Sigma-Aldrich | T6793 |
| Rabbit anti-acetylated tubulin | Cell Signaling Technology | 5335 |
| Mouse anti-α-Tubulin | Sigma-Aldrich | T9026 |
| Mouse anti-HA-tag | MBL | M180-3 |
| Mouse anti-FLAG-tag | Sigma-Aldrich | F3165 |
| Rabbit anti-HA-tag | Sigma-Aldrich | H6908 |
| Rabbit anti-FLAG-tag | MBL | PM020 |
| Mouse anti-DRC1 | This paper | N/A |
| Mouse anti-DRC2 | This paper | N/A |
| Mouse anti-DRC3 | This paper | N/A |
| Mouse anti-DRC4 | This paper | N/A |
| Mouse anti-DRC7 | This paper | N/A |
| Rabbit anti-AKAP3 | Proteintech | 13907-1-AP |
| Mouse anti-AKAP82/AKAP4 | BD Biosciences | 611564 |
| Mouse anti-PKAα cat | Santa Cruz Biotechnology | sc-28315 |
| Rabbit anti-PKA R1 | Abclonal | A0906 |
| Rabbit anti-PKA R2 | Abcam | Ab38949 |
| Rabbit anti-Luciferase | Proteintech | 27986-1-AP |
| Rabbit anti- Phospho-Tyrosine | PTMBIO | PTM-702RM |
| Goat anti-Rabbit IgG, HRP | Invitrogen | 31460 |
| Goat anti-Mouse IgG, HRP | Invitrogen | 31430 |
| Donkey anti-Mouse IgG, Alexa Fluor 488 | Invitrogen | A21202 |
| Donkey anti-Rabbit IgG, Alexa Fluor 488 | Invitrogen | A21206 |
| Donkey anti-Mouse IgG, Alexa Fluor 555 | Invitrogen | A31570 |
| Donkey anti-Rabbit IgG, Alexa Fluor 555 | Invitrogen | A31572 |
| **Bacterial and virus strains** | | |
| *E.coli* DH5a competent cells | Vazyme | C502-03 |
| *E.coli* strain BL21 (DE3) | Vazyme | C504-03 |
| **Chemicals, peptides, and recombinant proteins** | | |
| Glutaraldehyde,2.5% | Solarbio | P1126 |
| 4% paraformaldehyde | Beyotime | P0099 |
| RIPA | Beyotime | P0013C |
| Ni-NTA His Bind Resin | TransGen Biotech | DP101-01 |
| HTF | Irvine Scientific | 90126 |
| Hoechst 33342 | Sigma-aldrich | H6024 |
| PNA | Vectorlabs | RL-1072 |
| Lipofectamine 2000 | Invitrogen | 11668019 |
| **Critical commercial assays** | | |
| FastPure Gel DNA Extraction Mini Kit | Vazyme | DC301 |
| EndoFree Plasmid MidiKit | Cwbio | cw2105s |
| Periodic Acid Schiff (PAS) Stain Kit | Solarbio | G1280 |
| Pierce crosslink IP kit | Thermo Fisher | 26147 |
| FluoSpheres Fluorescent Microspheres | Thermo Fisher | F8813 |
| **Deposited data** | | |
| Differential proteome in WT and *Drc4* KO testis via anti-DRC4 antibody | This paper | N/A |
| **Experimental models: Cell lines** | | |
| HEK293T cell | Enogene | EGC238 |
| **Experimental models: Organisms/strains** | | |
| Mouse: C57BL/6J | Animal Core Facility of Nanjing Medical University | N/A |
| Mouse: ICR | Animal Core Facility of Nanjing Medical University | N/A |
| Mouse: *Drc1* KO | This paper | N/A |
| Mouse: *Drc2* KO | This paper | N/A |
| Mouse: *Drc3* KO | This paper | N/A |
| Mouse: *Drc4* KO | This paper | N/A |
| Mouse: *Drc7* KO | This paper | N/A |
| **Recombinant DNA** | | |
| Plasmid: pCAG1.1-Akap3-FLAG | This paper | N/A |
| Plasmid: pCAG1.1-Drc4-HA | This paper | N/A |
| Plasmid: pCAG1.1-Drc4-△(1-258)-HA | This paper | N/A |
| Plasmid: pCAG1.1-Drc4-△(272-392)-HA | This paper | N/A |
| Plasmid: pCAG1.1-Drc4-△(396-478)-HA | This paper | N/A |
| Plasmid: pCAG1.1-Drc2-HA | This paper | N/A |
| Plasmid: pCAG1.1-Drc1-FLAG | This paper | N/A |
| Plasmid: psiCHECK2-luciferase | This paper | N/A |
| Plasmid: psiCHECK2-SV40-DRC1-FLAG-luciferase | This paper | N/A |
| Plasmid: psiCHECK2-CMV-DRC2-HA-luciferase | This paper | N/A |
| Plasmid: psiCHECK2-CMV-DRC2-HA-SV40-DRC1-FLAG-luciferase | This paper | N/A |
| Plasmid: pUC57-T7-sgRNA | Addgene | 51132 |
| Plasmid: pST1374-NLS-flag-linker-Cas9 | Addgene | 44758 |
| Plasmid: pET-28a (+)-Drc2-cDNA (aa 1–126) | This paper | N/A |
| Plasmid: pET-28a (+)-Drc4-cDNA (aa 1–478) | This paper | N/A |
| **Software and algorithms** | | |
| FIJI | FIJI Software | https://fiji.sc/ |
| GraphPad Prism version 8.3.0 | GraphPad Software | https://www.graphpad.com/scientific-software/prism/ |
| LAS X 3.7.1core | Leica, Wetzlar | N/A |
