# Supplementary material for: Core N-DRC components play a crucial role in embryonic development and postnatal organ development
Source: Cell Death Dis. 2025 Mar 15;16(1):176. doi: 10.1038/s41419-025-07506-2 (PMC11910659; doi:10.1038/s41419-025-07506-2)

Figure 1C

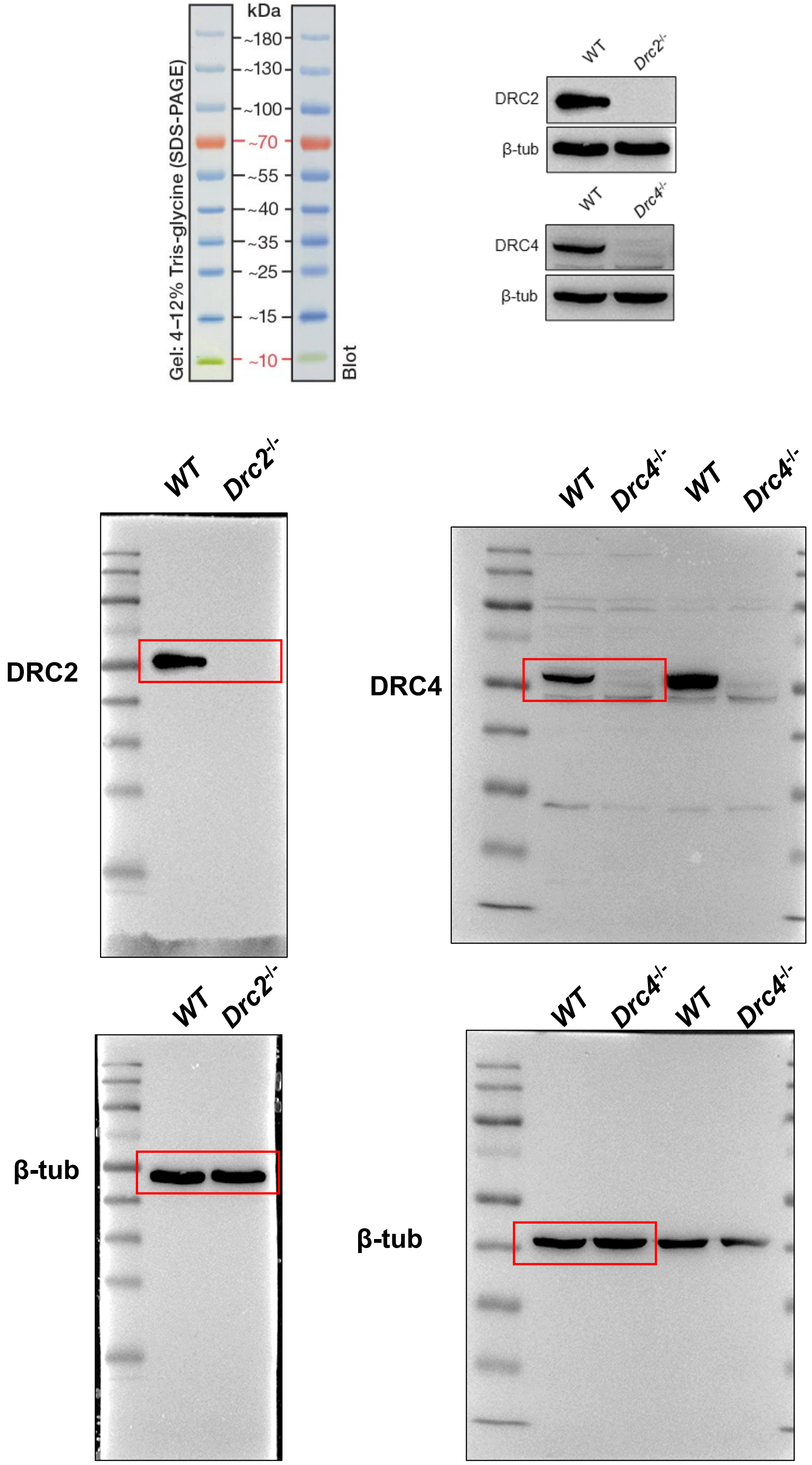
kDa
~180
~130
~100
~70
~55
~40
~35
~25
~15
~10
Gel: 4–12% Tris-glycine (SDS-PAGE)
Blot
WT
Drc2-/-
DRC2
β-tub
WT
Drc4-/-
DRC4
β-tub
WT
Drc2-/-
DRC2
WT
Drc4-/-
WT
Drc4-/-
DRC4
WT
Drc2-/-
β-tub
WT
Drc4-/-
WT
Drc4-/-
β-tub

Figure 5A

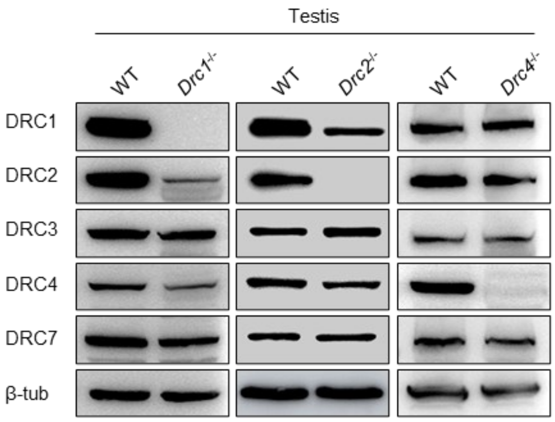

Testis
WT
Drc1-/-
WT
Drc2-/-
WT
Drc4-/-
DRC1
DRC2
DRC3
DRC4
DRC7
β-tub

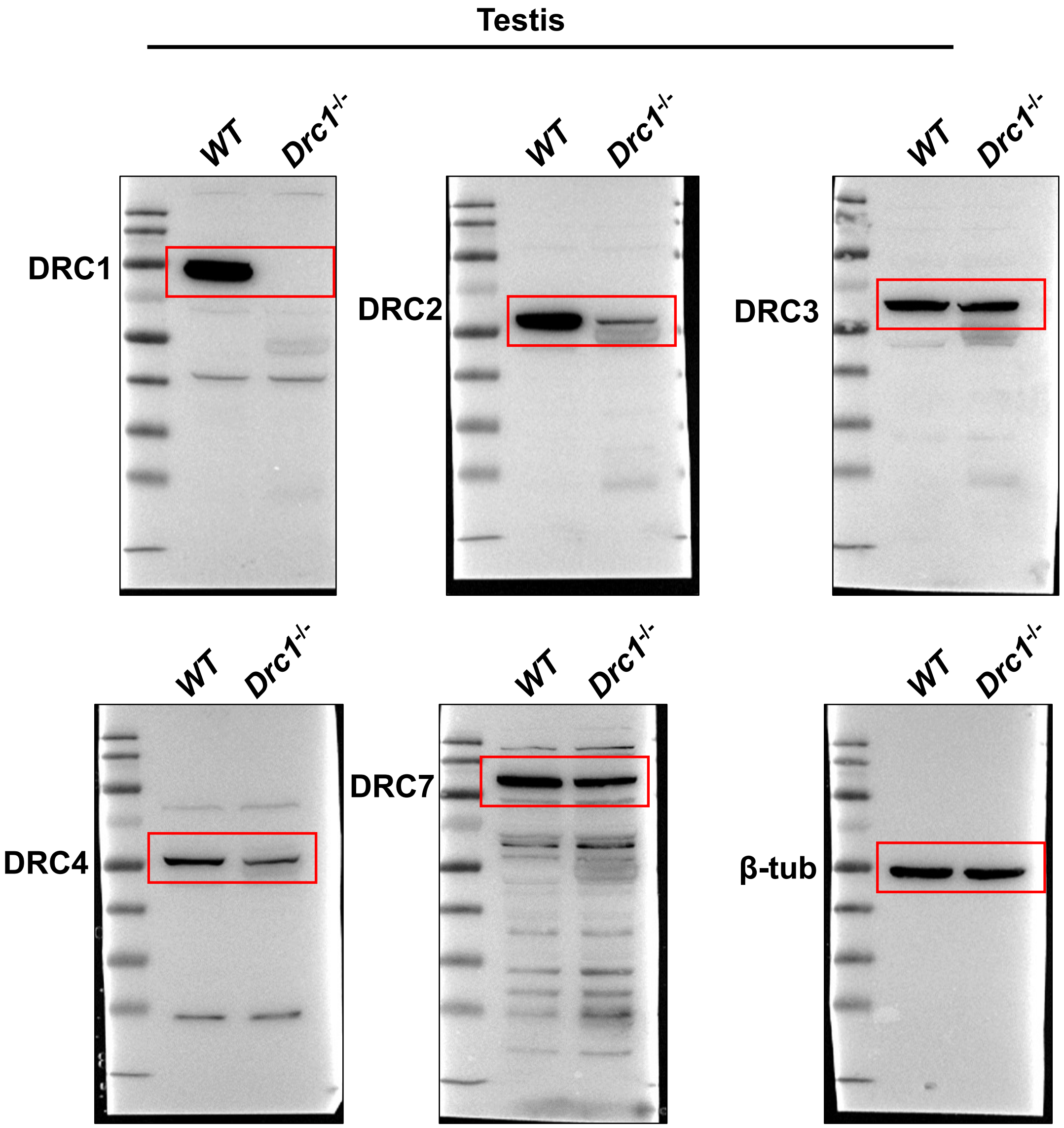

Testis
WT
Drc1-/-
DRC1
DRC2
DRC3
DRC4
DRC7
β-tub

Figure 5A

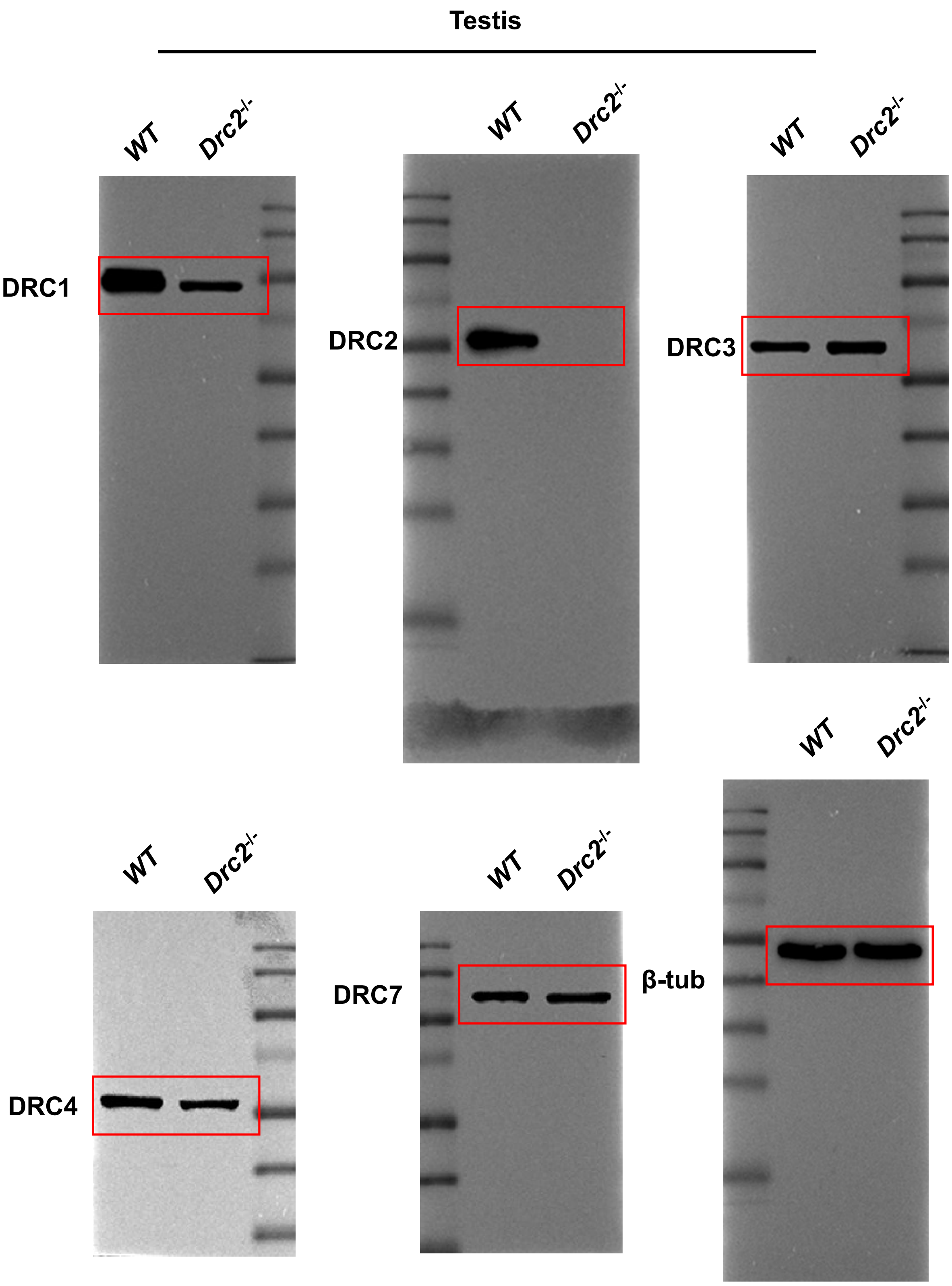

Figure 5A

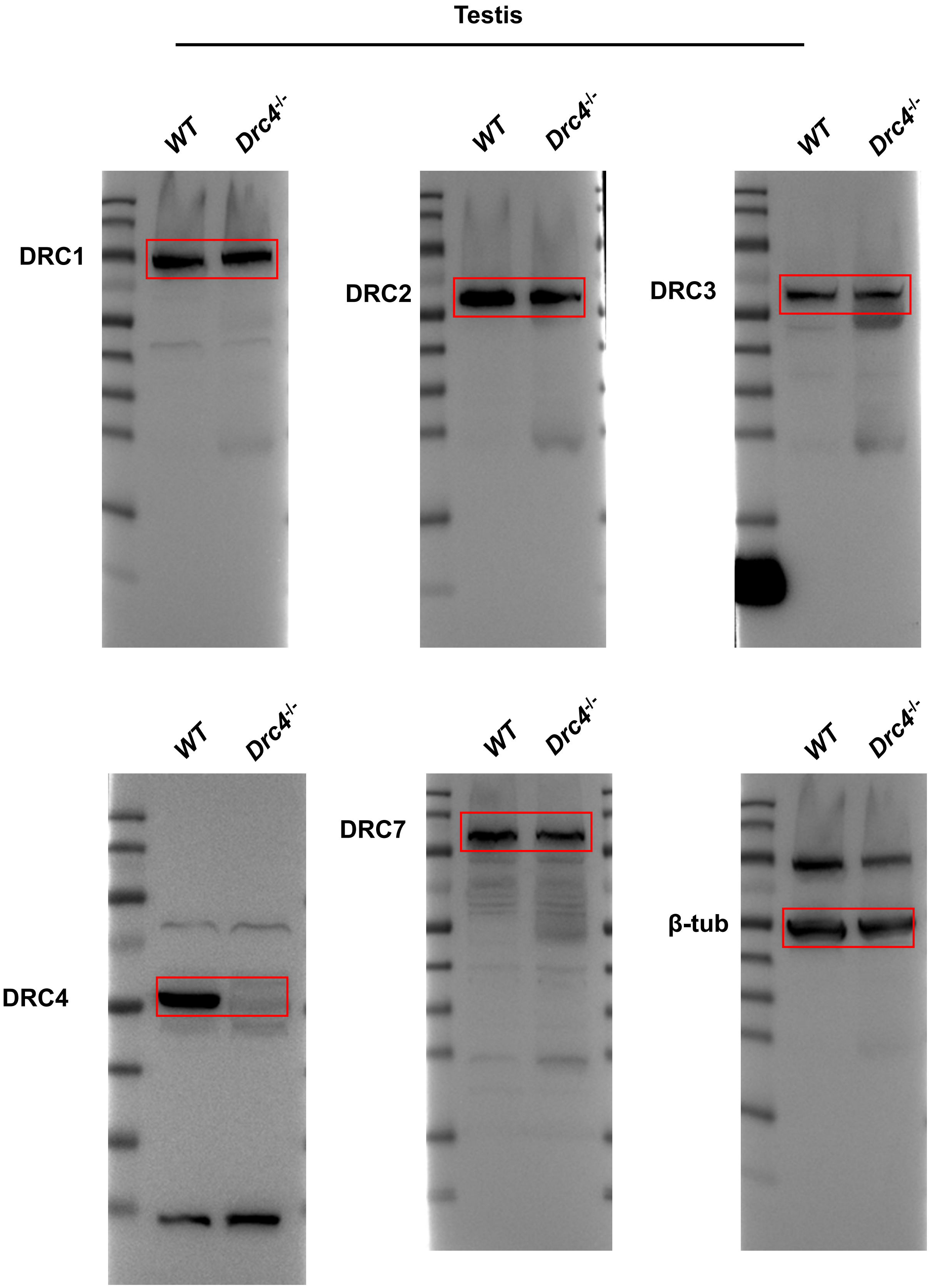

Figure 5B

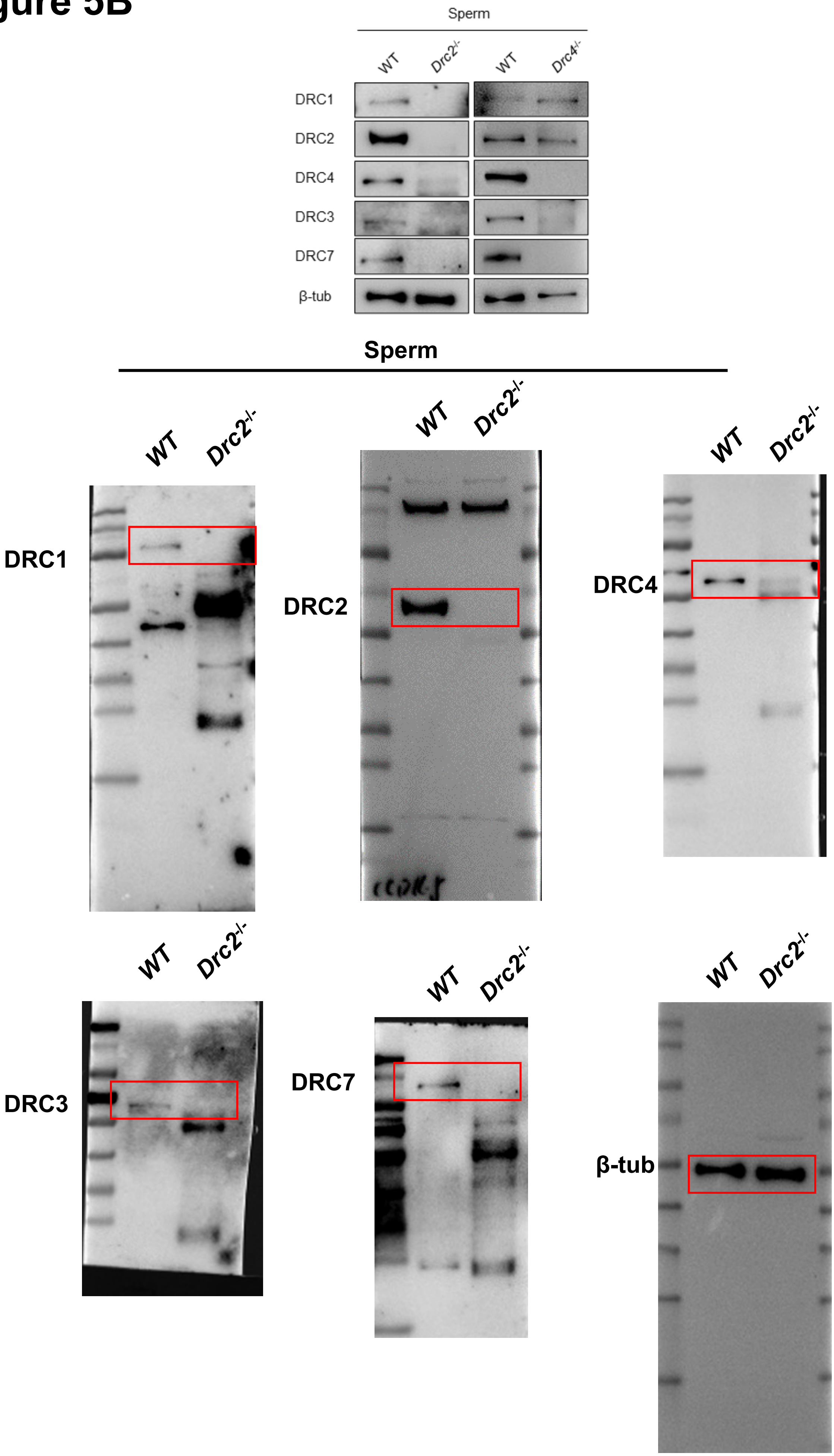
Sperm
WT
Drc2-/-
WT
Drc4-/-
DRC1
DRC2
DRC4
DRC3
DRC7
β-tub
Sperm
WT
Drc2-/-
DRC1
DRC2
DRC4
DRC3
DRC7
β-tub

Figure 5B

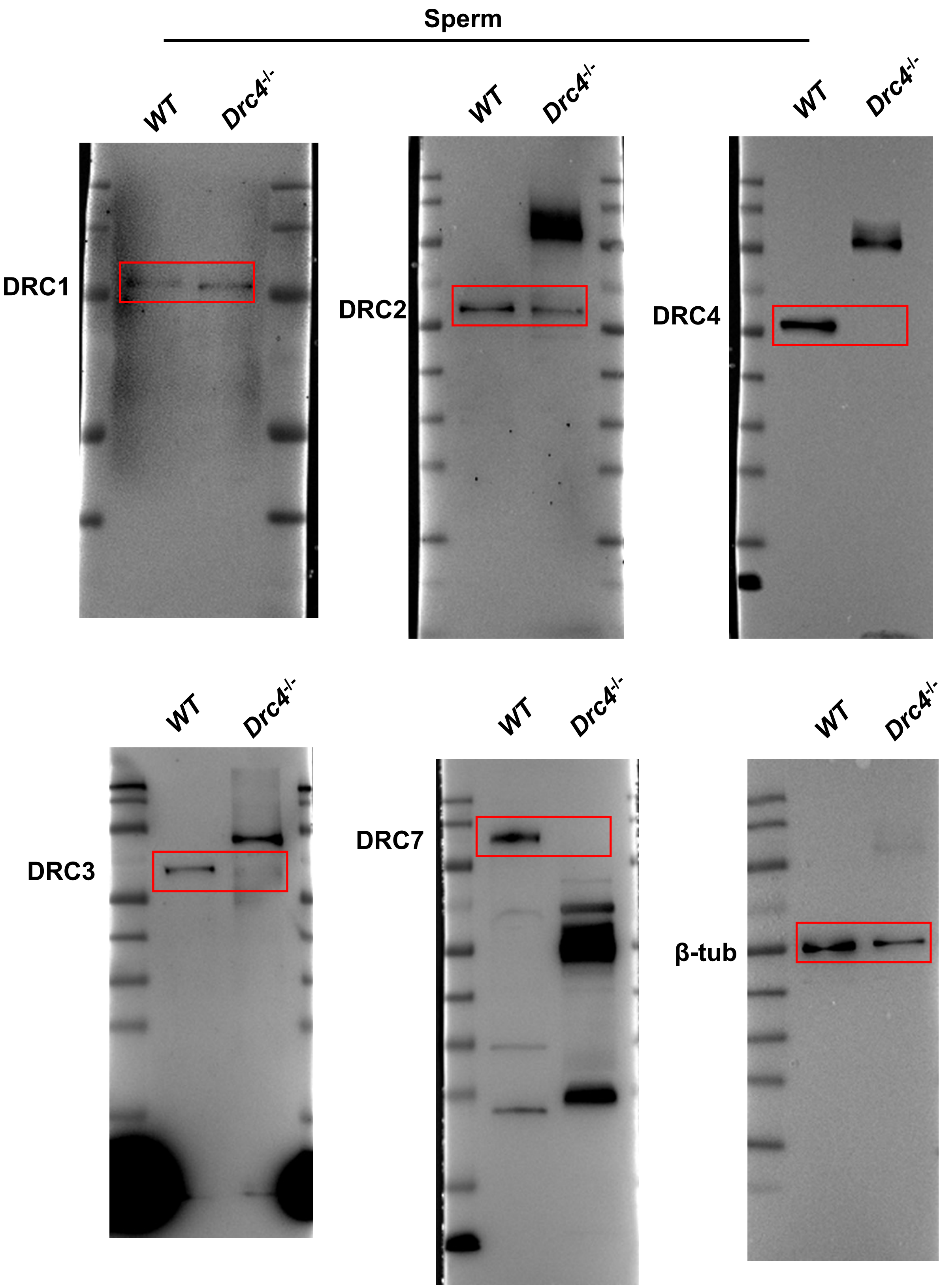

Figure 6A

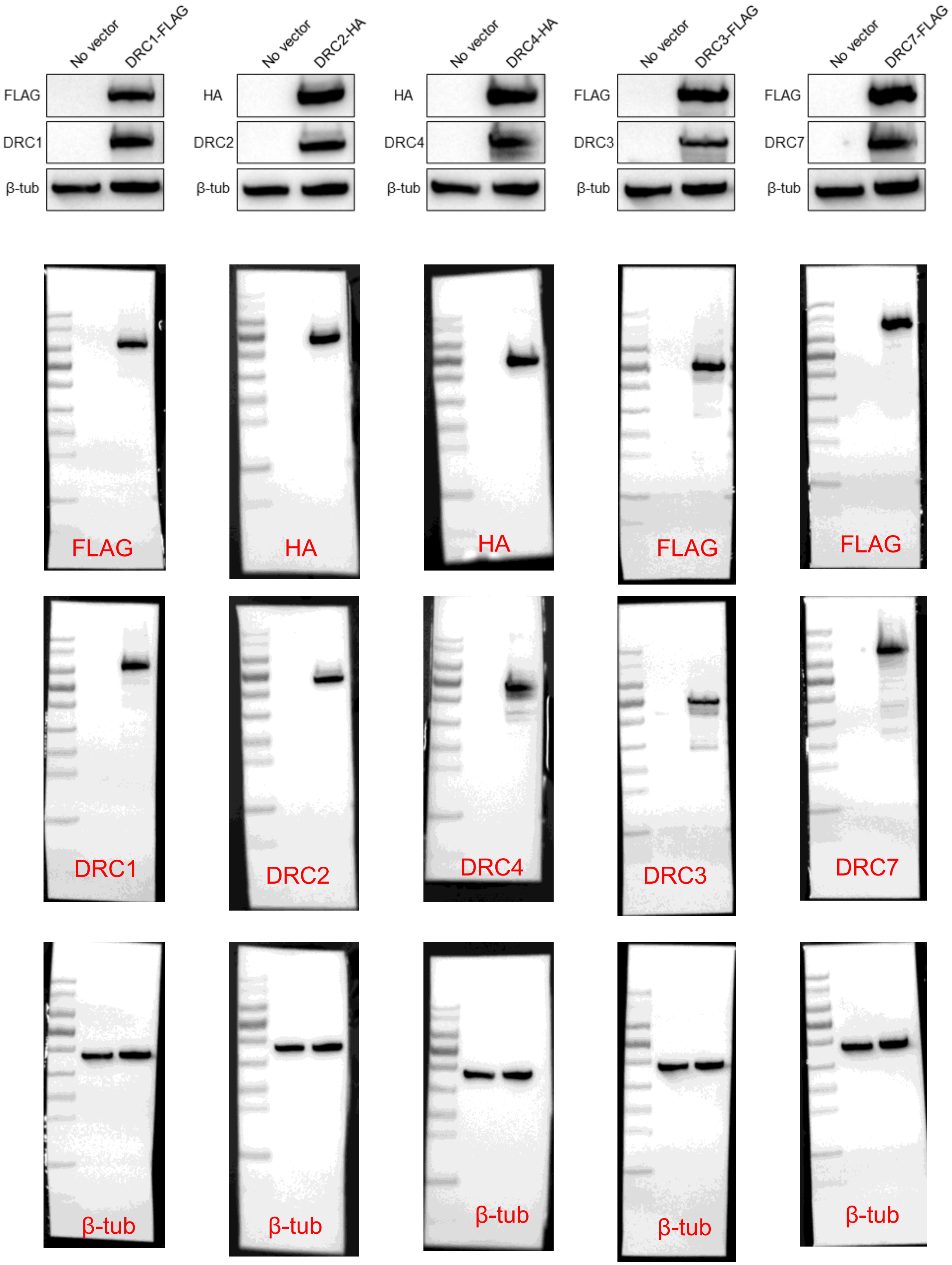

No vector
DRC1-FLAG
DRC2-HA
DRC4-HA
DRC3-FLAG
DRC7-FLAG
FLAG
HA
DRC1
DRC2
DRC4
DRC3
DRC7
β-tub

Figure 7A

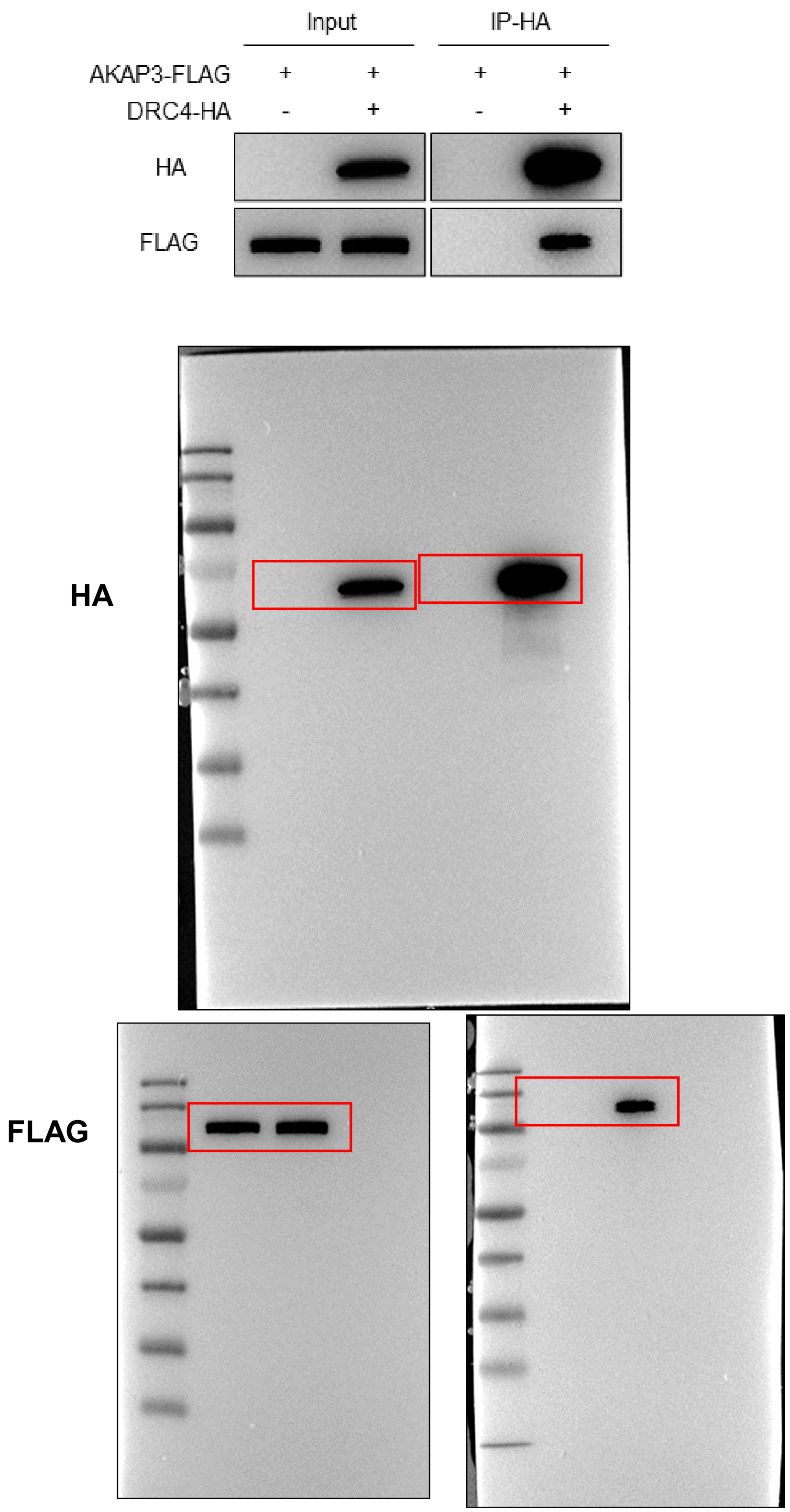

Figure 7B

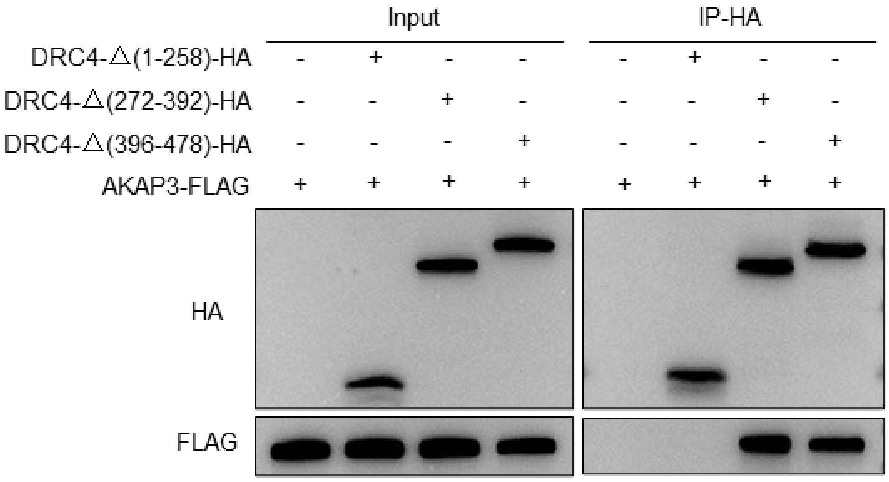

Input
IP-HA
DRC4-△(1-258)-HA
DRC4-△(272-392)-HA
DRC4-△(396-478)-HA
AKAP3-FLAG
HA
FLAG

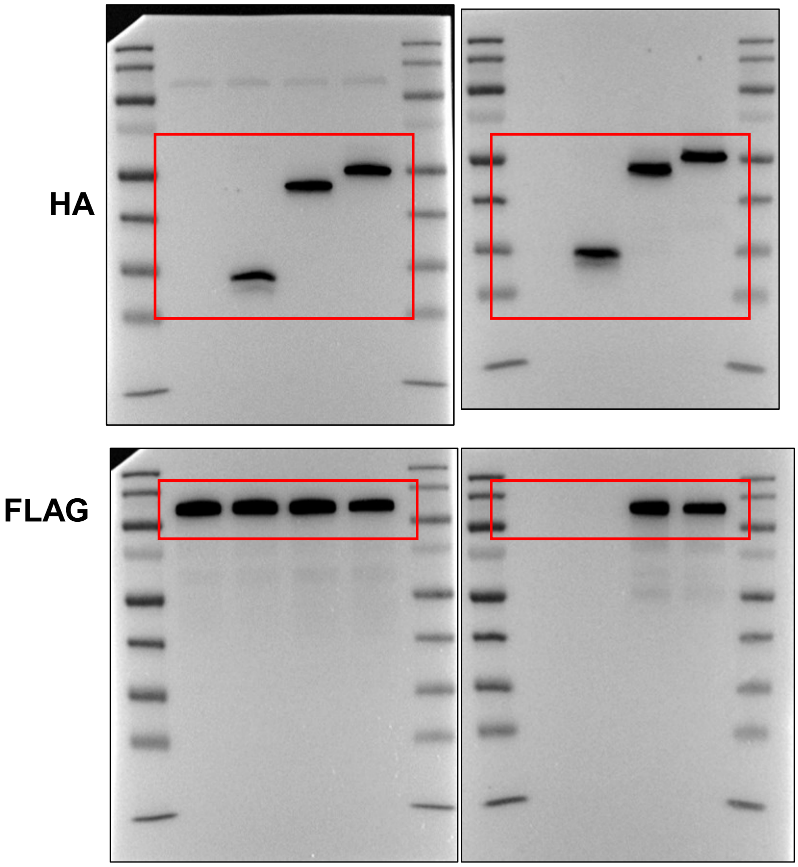

HA
FLAG

Figure 7C

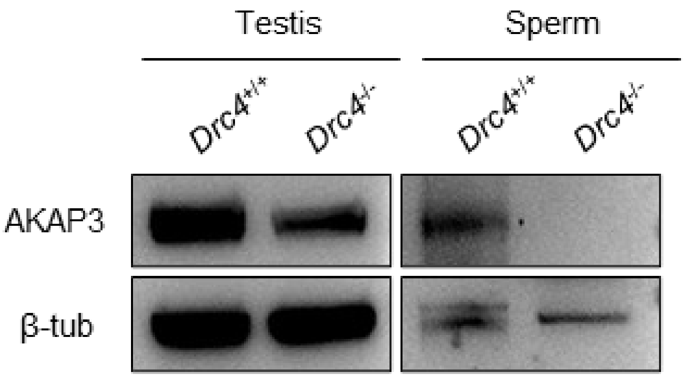

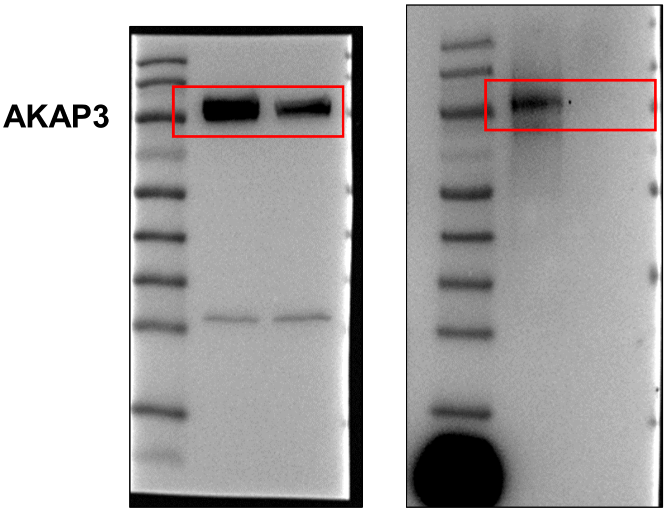

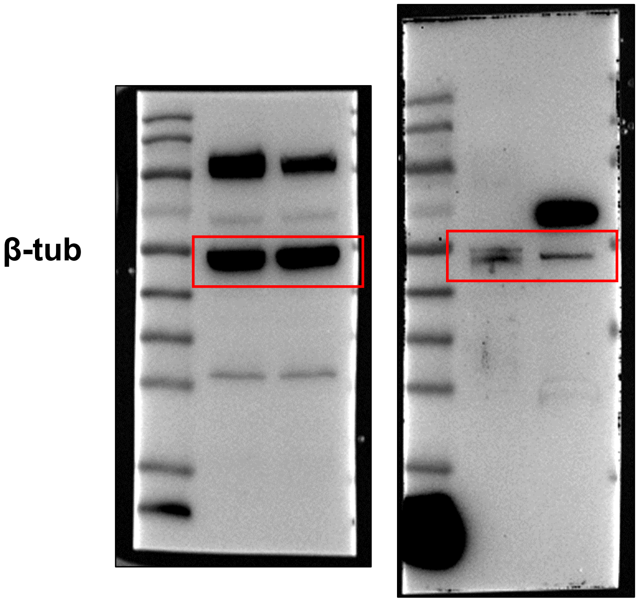

Figure 7G

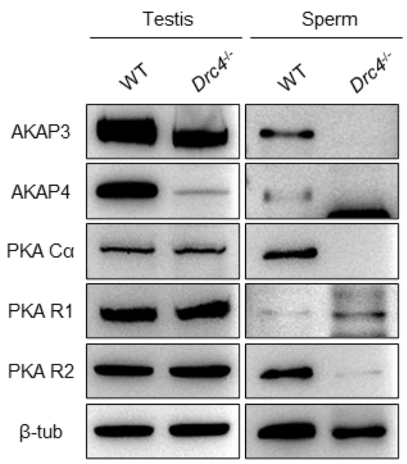
Testis
Sperm
WT
Drc4-/-
WT
Drc4-/-
AKAP3
AKAP4
PKA Cα
PKA R1
PKA R2
β-tub

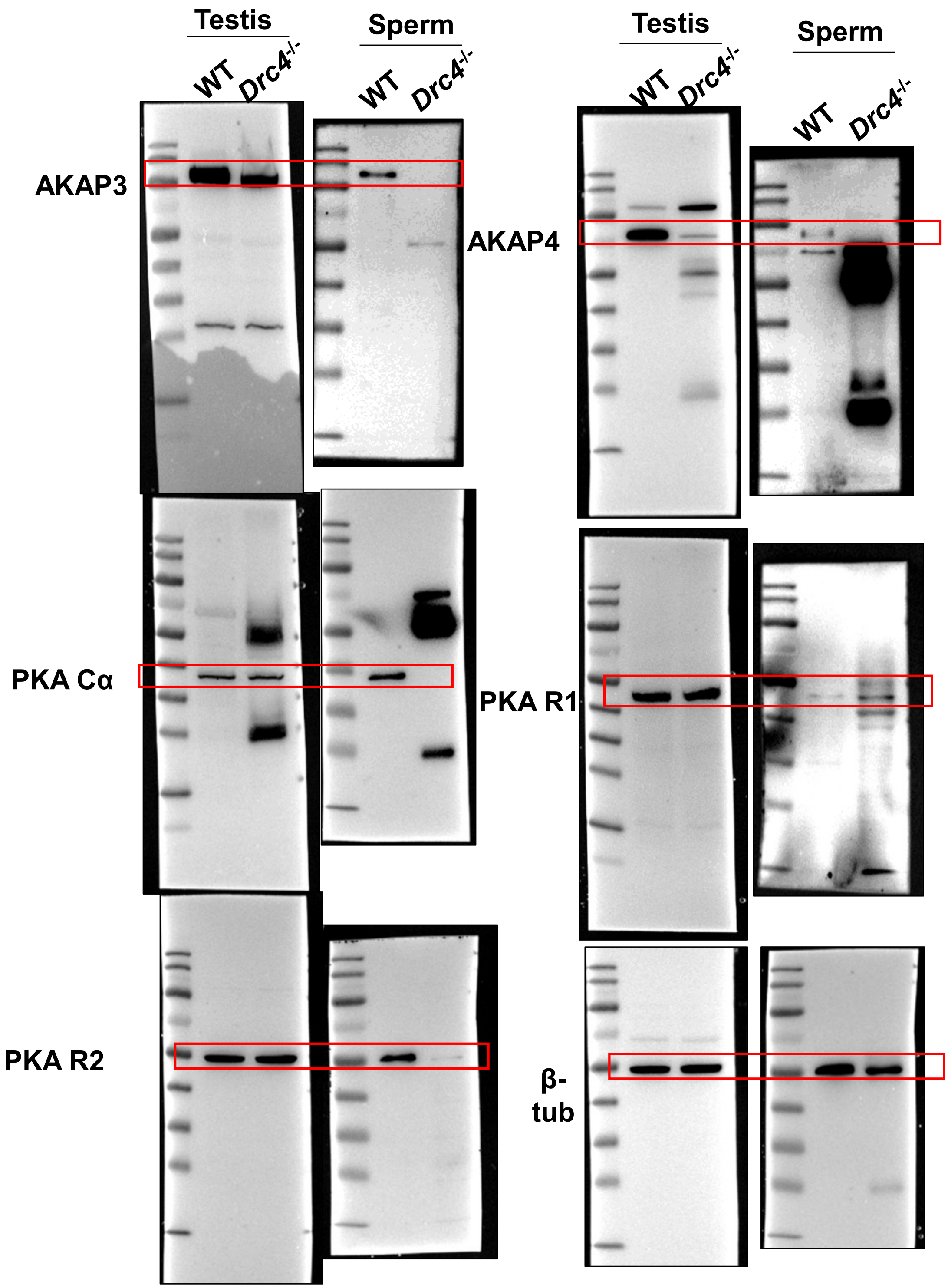
Testis
Sperm
WT
Drc4-/-
WT
Drc4-/-
Testis
Sperm
WT
Drc4-/-
WT
Drc4-/-
AKAP3
AKAP4
PKA Cα
PKA R1
PKA R2
β-tub

Figure 7H

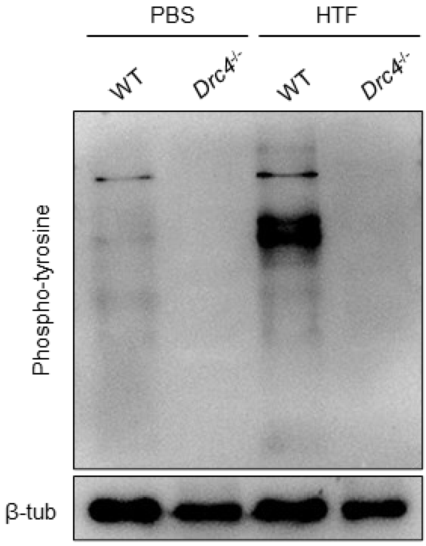
PBS
HTF
WT
Drc4-/-
WT
Drc4-/-
Phospho-tyrosine
β-tub

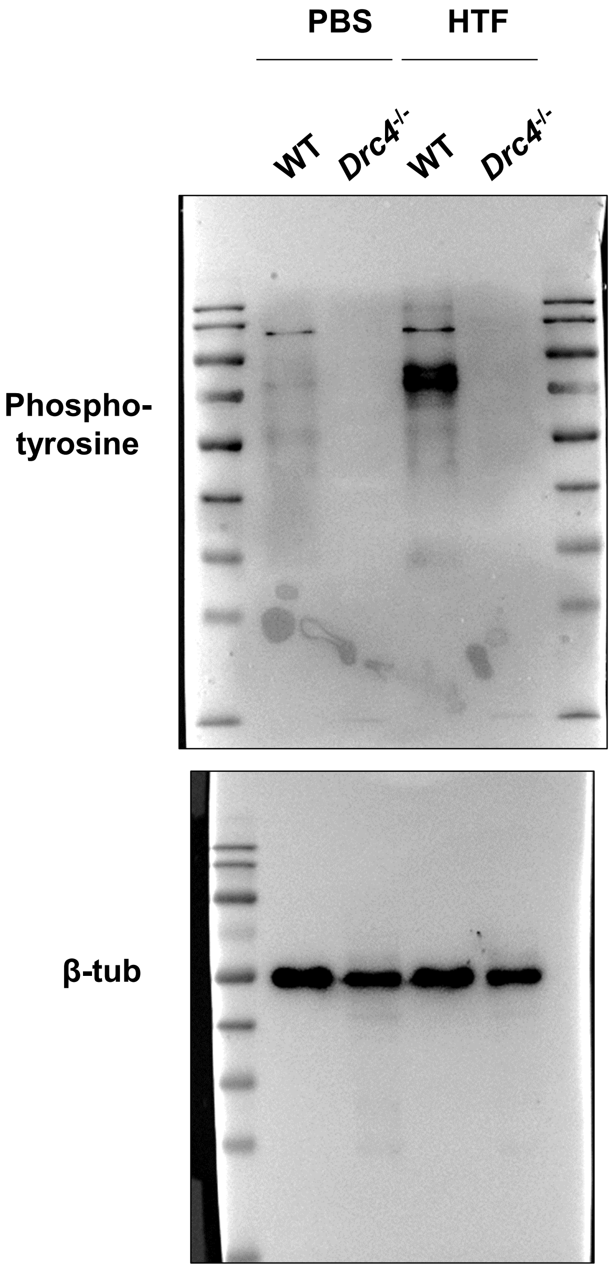
PBS
HTF
WT
Drc4-/-
WT
Drc4-/-
Phospho-tyrosine
β-tub

Supplementary Figure 3A

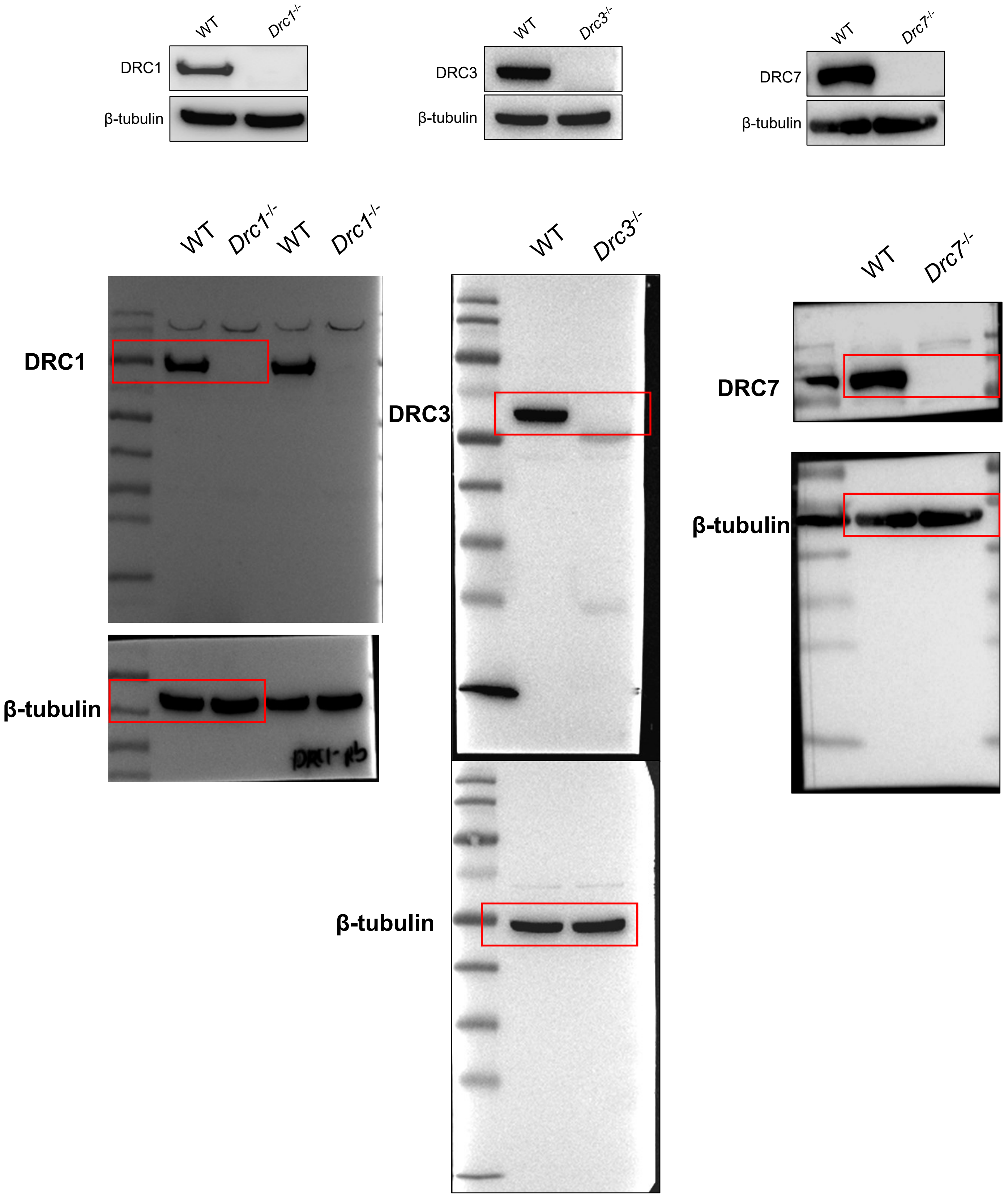

Supplementary Figure 4A

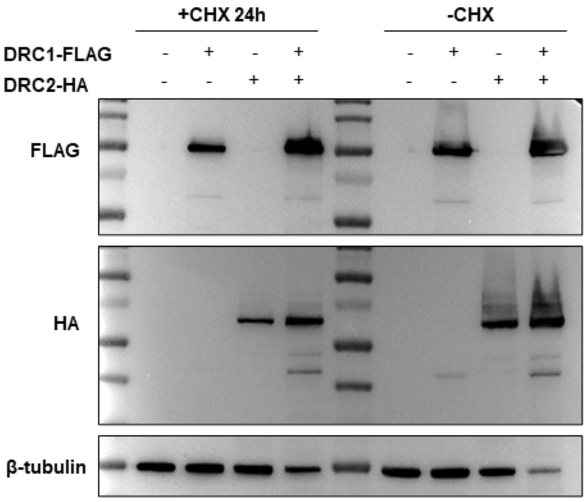

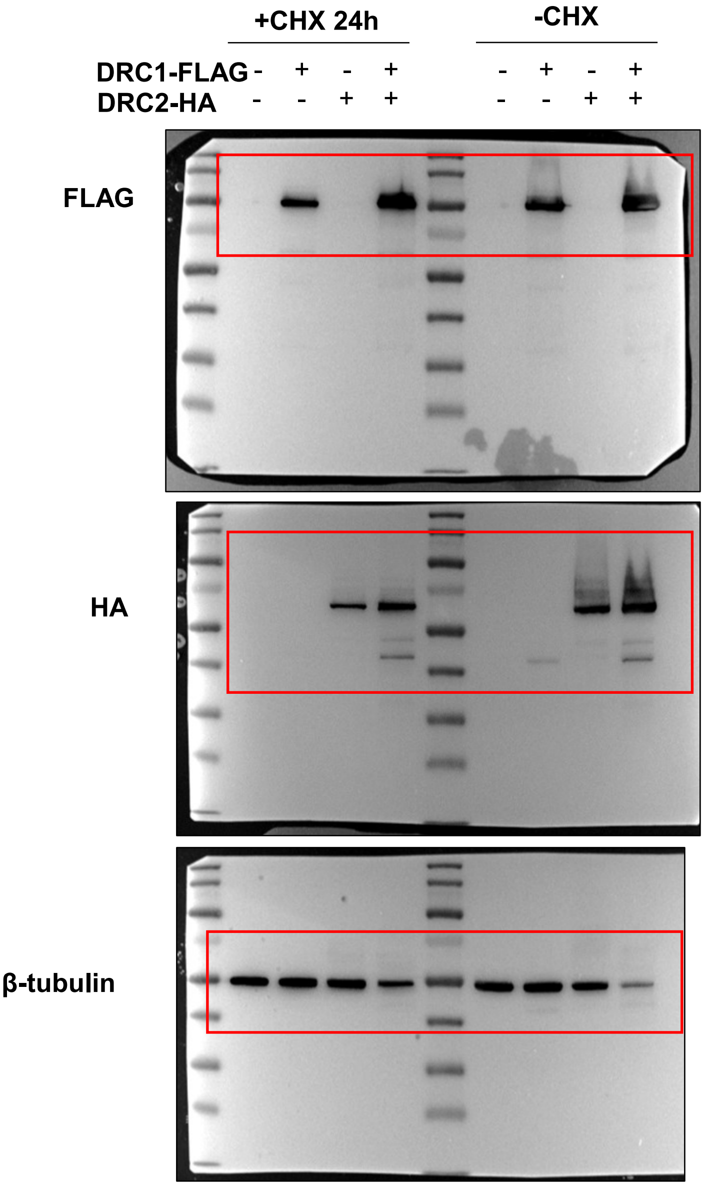

Supplementary Figure 4B

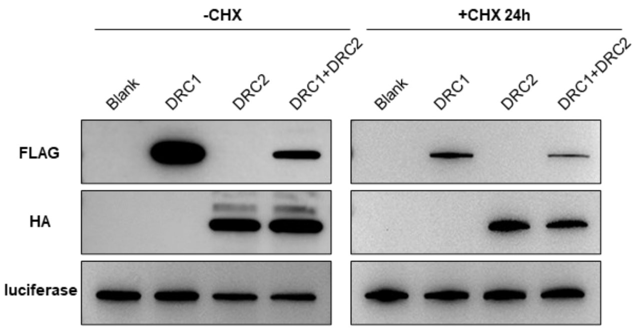

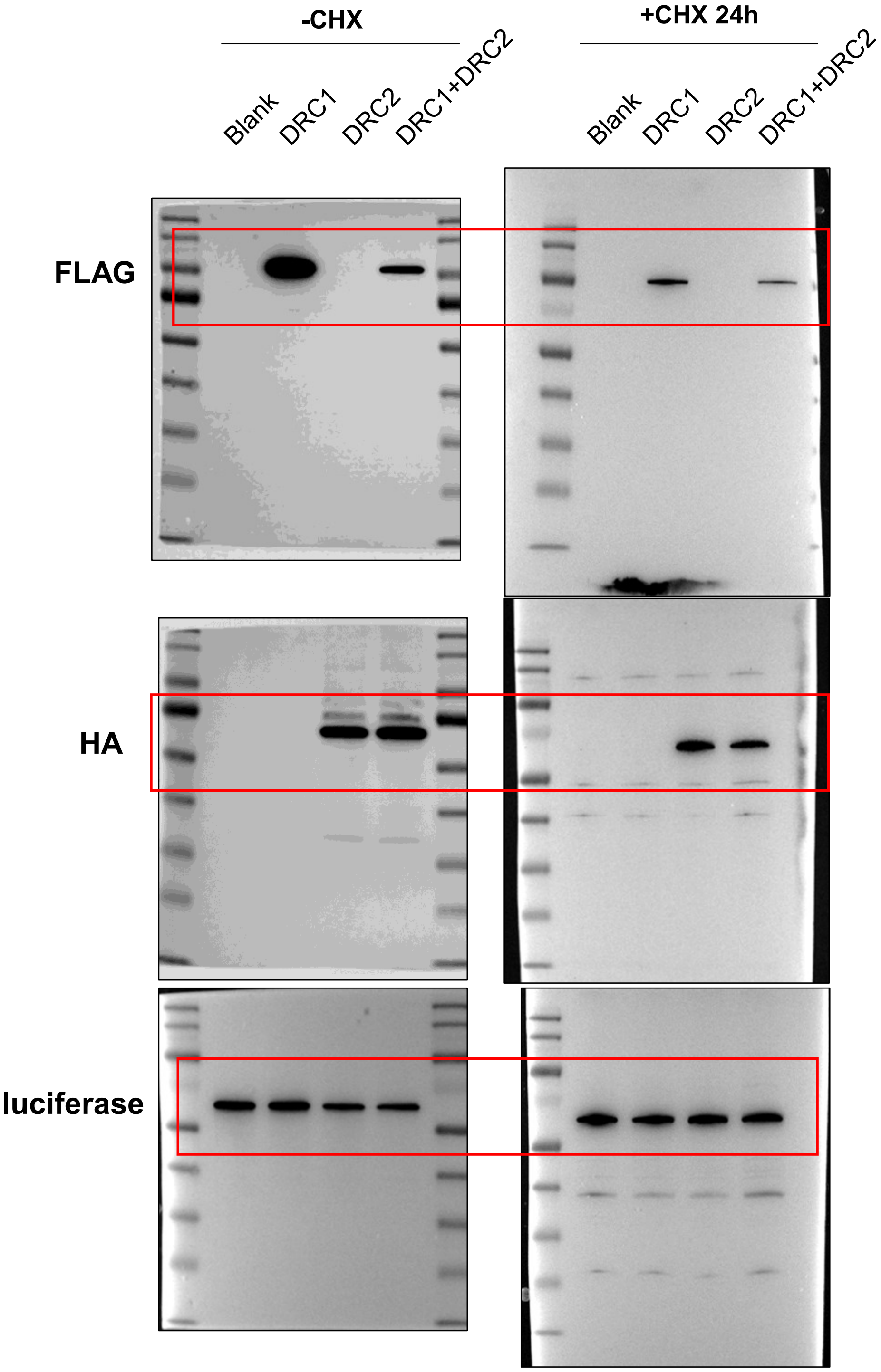

Supplement: Supplementary file 3 — Full and uncropped western blots [file 41419_2025_7506_MOESM3_ESM.pdf]
